# Comparative Subsequence Sets Analysis (CoSSA) is a robust approach to identify haplotype specific SNPs; mapping and pedigree analysis of a potato wart disease resistance gene *Sen3*

**DOI:** 10.1186/s13007-019-0445-5

**Published:** 2019-05-29

**Authors:** Charlotte Prodhomme, Danny Esselink, Theo Borm, Richard G. F. Visser, Herman J. van Eck, Jack H. Vossen

**Affiliations:** Wageningen UR Plant Breeding, Droevendaalsesteeg 1, 6708 PB Wageningen, The Netherlands

**Keywords:** Bulked segregant analysis (BSA), Whole genome sequencing (WGS), Reference genome, *k*-mers, Diagnostic markers, Potato wart disease

## Abstract

**Background:**

Standard strategies to identify genomic regions involved in a specific trait variation are often limited by time and resource consuming genotyping methods. Other limiting pre-requisites are the phenotyping of large segregating populations or of diversity panels and the availability and quality of a closely related reference genome. To overcome these limitations, we designed efficient Comparative Subsequence Sets Analysis (CoSSA) workflows to identify haplotype specific SNPs linked to a trait of interest from Whole Genome Sequencing data.

**Results:**

As a model, we used the resistance to *Synchytrium endobioticum* pathotypes 2, 6 and 18 that co-segregated in a tetraploid full sib population. Genomic DNA from both parents, pedigree genotypes, unrelated potato varieties lacking the wart resistance traits and pools of resistant and susceptible siblings were sequenced. Set algebra and depth filtering of subsequences (*k*-mers) were used to delete unlinked and common SNPs and to enrich for SNPs from the haplotype(s) harboring the resistance gene(s). Using CoSSA, we identified a major and a minor effect locus. Upon comparison to the reference genome, it was inferred that the major resistance locus, referred to as *Sen3*, was located on the north arm of chromosome 11 between 1,259,552 and 1,519,485 bp. Furthermore, we could anchor the unanchored superscaffold DMB734 from the potato reference genome to a synthenous interval. CoSSA was also successful in identifying *Sen3* in a reference genome independent way thanks to the de novo assembly of paired end reads matching haplotype specific *k*-mers. The de novo assembly provided more R haplotype specific polymorphisms than the reference genome corresponding region. CoSSA also offers possibilities for pedigree analysis. The origin of *Sen3* was traced back until Ora. Finally, the diagnostic power of the haplotype specific markers was shown using a panel of 56 tetraploid varieties.

**Conclusions:**

CoSSA is an efficient, robust and versatile set of workflows for the genetic analysis of a trait of interest using WGS data. Because the WGS data are used without intermediate reads mapping, CoSSA does not require the use of a reference genome. This approach allowed the identification of *Sen3* and the design of haplotype specific, diagnostic markers.

**Electronic supplementary material:**

The online version of this article (10.1186/s13007-019-0445-5) contains supplementary material, which is available to authorized users.

## Background

The identification of genetic loci involved in important traits variation is a fundamental step in genetic research and breeding. QTL mapping strategies are often time-consuming and laborious. Often, large biparental populations (n > 300) segregating for the trait of interest are used, which necessitates considerable efforts for genotyping and phenotyping. Genome-Wide Association Studies (GWAS) are alternatives but equally require genotyping and phenotyping of a broad panel of individuals. Bulked Segregant Analysis (BSA) [1, 2], simplify the genotyping effort as pools of individuals with contrasting phenotypes are used, which drastically reduces the number of samples to be genotyped. BSA consists of estimating and comparing allelic frequencies between the pools. The frequency of non-linked loci for the trait of interest are expected to be equivalent between the pools whereas a bias in the frequency of the loci linked to the causal genes is expected. This way, DNA sequence variants linked to the trait of interest are identified, which allows the development of markers for genetic mapping and Marker Assisted Selection (MAS) purposes.

A conventional BSA approach uses a set of formerly identified markers [2, 3] such as SNP-arrays [4]. Using markers not specifically developed for the population of interest can lead to ascertainment bias resulting in a limited number of markers, which may result in low mapping resolution. In polyploids, ascertainment bias is even a more severe problem, as markers in linkage disequilibrium are less informative. As sequencing became more affordable, genotyping-by-sequencing (GBS) methods, such as whole genome sequencing (WGS) [5], RNA-Seq [6] or different complexity reduction strategies [7, 8] have been applied in combination with BSA. These BSA methods, collectively referred to as mapping by sequencing (MBS) [9], involve the successive extraction, pooling and sequencing of the DNA of the pooled individuals. The reads obtained from these bulks are mapped to a reference genome and variants are called in each bulk. The allelic frequencies of the variants are compared between the bulks and variants in linkage disequilibrium with the locus encoding the trait of interest are identified. When WGS data are used, the resolution is increased and it becomes possible to not only identify polymorphisms in linkage disequilibrium but also in the causal locus itself [5].

Methods using read mapping rely on the availability of a reference genome. Nowadays, many crops have reference genomes available, but this is not yet the case for some non-model species or complex polyploid species. Even if a reference genome is available for the crop of interest, the assembly quality or completeness of the reference can be sub-optimal. Moreover, the genotype used as a reference can be considerably different from the genetic background of the population of interest. The wheat pan-genome was studied by comparing whole genome sequencing data of 18 different cultivars [10]. Interestingly, only 64% of the genes were present in all the cultivars and the rest showed presence-absence variations between them. This study illustrates why reference genome based approaches for genetic analysis and crop improvement can cause limited and incomplete results. This limitation is even more prominent in the case of disease resistance genes, which reside in clusters of highly similar paralogous sequences inherently difficult to assemble and highly variable in organization. Moreover, the genotype used as a reference is often susceptible to the disease and lacks the causal *R* gene paralog segregating in a mapping population. This is exemplified by the case of the fine-mapping and candidate gene analysis of a powdery mildew resistance QTL in barley [11]. After an exome capture experiment was performed using homozygous recombinants, reads were mapped to the barley reference genome. Surprisingly, heterozygous sites were observed in genomic regions containing *R* genes. This could be explained by assuming the reference genome is missing some paralogs of an *R* gene cluster. The reads from the recombinant lines coming from these missing paralogs wrongly mapped to similar paralogs. In this case, a de novo assembly of the wrongly mapped reads was needed to identify the candidate genes.

Working directly with WGS reads without alignment to a reference offers several advantages. The use of subsequences (*k*-mers of *k* nucleotides) instead of the full reads allows to bypass sequencing errors in the reads and allows unambiguous comparisons between samples thanks to their invariable length. A notable example of the use of *k*-mers is provided in a study aiming to identify variants associated with human diseases [12]. Here, the authors identified several disease-associated sequences that were not present in the human reference genome. A similar approach was applied in a panel of wheat accessions [13]. The authors developed a new method, AgRenSeq, which combines resistance gene enrichment (RenSeq) and a *k*-mer based GWAS. They phenotyped an *Aegilops tauschii* panel with different stem rust races and intended to identify stem rust resistance (*Sr*) genes. AgRenSeq was successfully applied to identify and clone four functional *R* genes independently from a reference genome in a diversity panel.

In this paper, we describe a set of workflows including a BSA approach using *k*-mers instead of variants. The Comparative Subsequence Sets Analysis (CoSSA) is suitable to quickly identify haplotype specific SNPs to develop diagnostic markers linked to traits of interest, with or without a reference genome. As an example, we used a tetraploid potato (*Solanum tuberosum*) population segregating for potato wart disease resistance.

Potato wart disease is caused by the obligate biotrophic soil-borne *Synchytrium endobioticum* Chytridiomycete fungus, which can result in dramatic yield losses. This fungus produces spores that can persist in the soil for more than 40 years [14]. In addition, no chemicals are available to control the pathogen. For these reasons, quarantine regulations are imposed [15], forbidding the cultivation of potatoes on infested fields. In addition, in protection zones around the infested fields, only resistant varieties may be grown. Therefore, resistant varieties are an essential key to manage the disease. The *Sen1* locus providing resistance to pathotype 1 isolates has been mapped previously on the north arm of chromosome 11 [16]. Most potato varieties of the European gene pool are resistant to pathotype 1, but only a few are resistant to pathotypes 2, 6 and 18, all of which are occasionally detected in most European countries. Until recently, other QTLs giving resistance to various patterns of pathotypes have been mapped in diploid [17, 18] and tetraploid populations [19–21]. However, markers with diagnostic value were not found frequently. Only recently, a combined approach of linkage mapping, BSA and RNAseq data in a dihaploid potato population derived from the tetraploid variety Karolin allowed the identification of markers with diagnostic value linked to pathotypes 6 and 18 resistance on the north arm of chromosome 11 [22].

In this study, we applied the CoSSA workflows to map the *Synchytrium endobioticum* pathotype 2, 6 and 18 resistance from the variety Kuba, and refer to it as *Sen3.* CoSSA was implemented with and without the use of the potato reference genome and was successful in both cases. *Sen3* was fine-mapped and haplotype specific markers close to the resistance gene were developed and validated for Marker Assisted Selection. CoSSA also allowed us to improve the potato reference genome that is poorly assembled in the region of interest. Different aspects and further applications of CoSSA, such as pedigree analysis, are described in this paper and compared to earlier approaches. We found that *Sen3* descended from the variety Ora and was indistinguishable from the resistance in the variety Karolin [21, 22].

## Materials and methods

### Plant material and wart disease resistance phenotyping

The potato variety Kuba (resistant to the pathotypes 1, 2, 6, and 18 [23]) was crossed to the susceptible variety Ludmilla (resistant to the pathotype 1 only [23]), resulting in a full-sib tetraploid population (K*L) of 328 clones segregating for the pathotypes 2, 6 and 18 resistance (abbreviated as P2, P6 and P18 respectively).

Progeny clones and both parents were phenotyped in spring 2016 for potato wart disease resistance with the Glynne-Lemmerzahl [24, 25] method. For each progeny clone, five tubers were tested for pathotypes 2, 6 and 18 resistance. The isolates used for inoculation were respectively JKI P2(G1)-2009, JKI P6(O1)-2009 and JKI P18(T1)-2009. Resistance levels were scored from 1 (highly resistant, early defense necrosis) to 5 (highly susceptible, formation of small to large warts). Mean scores were calculated for each progeny clone (Additional file 1). Chi square tests were performed to analyze the resistance segregation ratio to the three tested pathotypes in the population.

To compose the susceptible bulk (S-bulk), 17 progeny clones fully susceptible to P2(G1), P6(O1) and P18(T1) in 2016 were selected. To compose the resistant bulk (R-bulk), 17 progeny clones that were fully resistant to the three pathotypes in 2016 were selected and were re-phenotyped (six tubers) in 2017. The three isolates and phenotyping method used were the same as the previous year. All the phenotypic assays were performed in the Laboratory of Quarantine Organisms, Department of Plant Pathology, IHAR-PIB, Poland.

### DNA extraction, pooling and sequencing

Genomic DNA of the bulked progeny clones, the two parents, the mother (Bzura), the suspected great-great-grand-father (BRA9089) of Kuba and of four susceptible varieties (Alegria, Desiree, Kuras and VR808) was extracted from freshly harvested leaves according to [26]. DNA concentration was assessed using a Qubit Fluorometer (Invitrogen). For the S-bulk and the R-bulk, 59 ng of DNA of each individual was pooled. For all WGS experiments, 1 µg of (pooled) genomic DNA was used for library preparation and sequenced on an Illumina HiSeq 2000 platform producing 151 bp paired end (PE) reads (Hartwig Medical foundation, Amsterdam, The Netherlands).

Genomic DNA of the entire full-sib population (n = 328) and of a panel of 56 resistant and susceptible potato varieties was extracted from freshly harvested leaves using a modified CTAB-method [27]. These varieties were selected to embody the different wart resistance sources present in the European breeding germplasm. The DNA concentration was estimated using a NanoDrop ND-1000 spectrophotometer (Thermo Scientific) and adjusted to a concentration of 5–50 ng/μl. The DNA quantity was confirmed and the quality was assessed on ethidium bromide containing agarose gels.

### Comparative subsequence sets analysis workflows

**Fig. 1:**
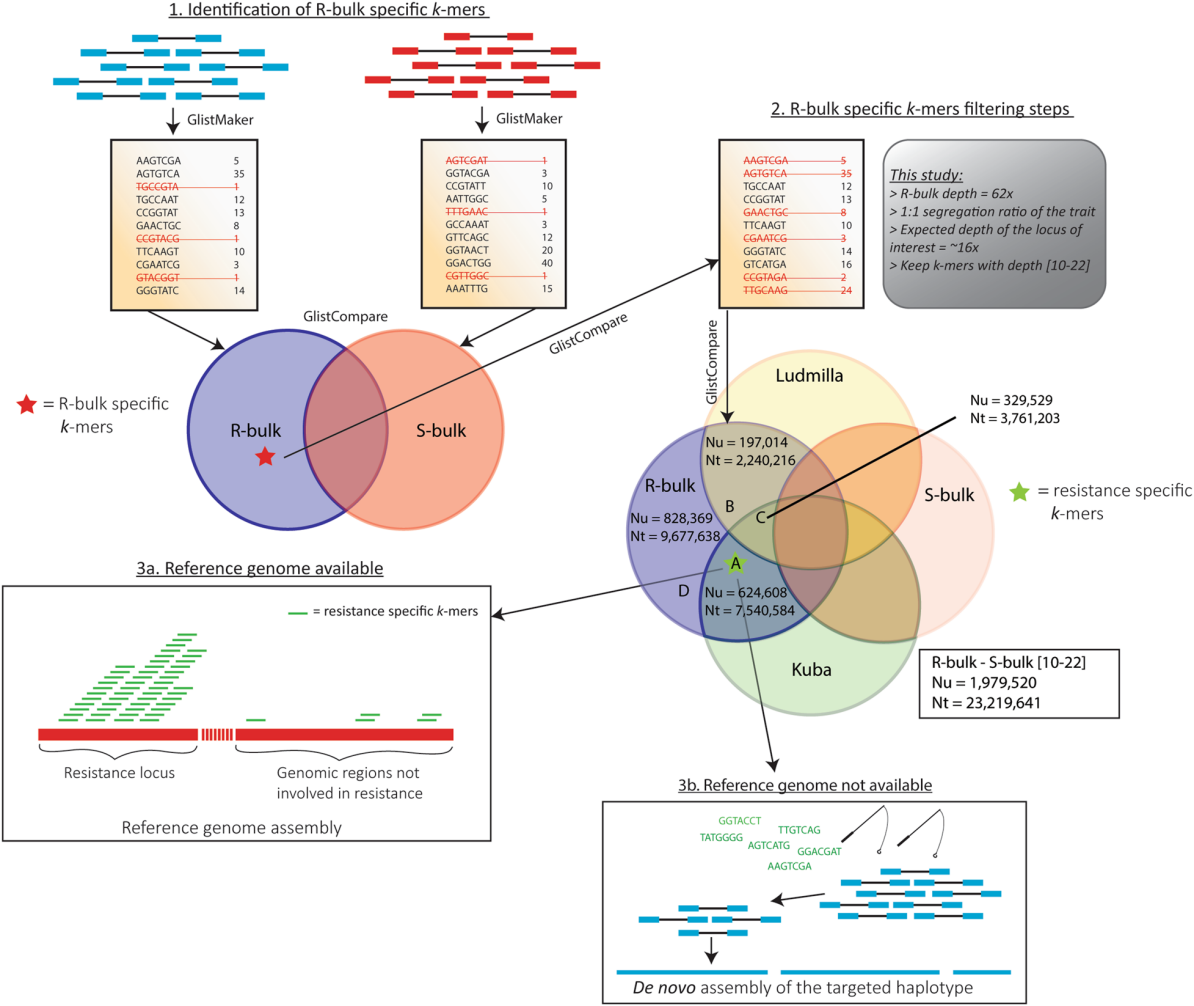
Comparative Subsequence Sets Analysis (CoSSA) workflow example. **1** The R-bulk, the S-bulk and (optionally) the parents of the full-sib population are sequenced with Illumina 151 bp PE. The Illumina reads are quality trimmed and *k*-mer tables are built for each of the samples. The *k*-mer tables contain all the possible *k*-mers of the sample and their frequency (~sequencing depth). *K*-mers with a frequency of 1 are considered as sequencing errors and are removed from the set. The difference between the R-bulk and S-bulk *k*-mer tables is performed to keep only the R-bulk specific *k*-mers. **2** R-bulk specific *k*-mers are filtered upon their depth in function of the expected depth of the R haplotype. In our situation, we worked with a tetraploid population in which the resistance segregated with a 1:1 segregation ratio. Each of the progeny clones pooled in the R-bulk held the R-haplotype in one copy (simplex) which means that 25% of the reads cover the R-haplotype. The depth of each haploid genome in our R-bulk was ~ 16 × so we kept the *k*-mers with a depth from 10 × to 22 ×. If the parents have been sequenced, additional set operations can be used to divide the *k*-mers in function of their parental origin: *k*-mers inherited from the resistant parent (Kuba, subset A: resistance specific *k*-mers), *k*-mers inherited from the susceptible parent (Ludmilla, subset B), *k*-mers inherited from both parents (subset C) and the *k*-mers inherited from none of the parents (subset D). **3a** If a reference genome is available, the *k*-mers are mapped to it to identify the resistance locus. **3b** If no reference genome is available, the read pairs containing the R-bulk specific *k*-mers are extracted from the raw reads data and used to re-assemble the resistant haplotype. Nu: Number of unique *k*-mers, Nt: Total number of *k*-mers

The CoSSA workflow used in this study is illustrated in Fig. 1. All the scripts, the software and the code used in CoSSA are available on GitHub (https://github.com/cprodhom/CoSSA-workflows). The computational requirements of each of the main steps of the workflows are given in Additional file 2. The forward and reverse reads were quality trimmed and Illumina adapters were removed using Trimmomatic version 0.32 [28] (settings ILLUMINACLIP:TruSeq3-PE.fa:2:30:10, LEADING:3, TRAILING:3, SLIDINGWINDOW:4:15 and MINLEN:70). *K*-mer tables were built for each sequenced sample by using the GlistMaker program of the GenomeTester4 toolkit [29]. GlistMaker generates *k*-mers lists from the trimmed reads and counts the frequency (≈ sequencing depth) of each unique *k*-mer. We used a *k*-mer size of 31 nucleotides. This *k*-mers size was chosen as a compromise between sequence uniqueness (increase the *k*-mer size increases the chances that each *k*-mer comes from a unique region of the genome) and sequence correctness (decrease the *k*-mer size allows to reduce the PE reads errors). The *k*-mers that were observed only once were removed from the dataset as they are likely caused by sequencing errors and would unnecessarily increase the required computational power and memory if retained. The GlistCompare program of GenomeTester4 was then used to perform basic set operations such as unions, intersections or differences.

To obtain *k*-mers exclusively present in the R-bulk (R-bulk specific *k*-mers) we subtracted the S-bulk *k*-mers from the R-bulk. Additional filtering steps were applied to refine the R-bulk specific *k*-mers selection. The sequencing yield was 52 Gb for the R bulk. Therefore, the sequencing depth of the R-bulk was approximately 62× considering a haploid genome of 840 Mb. Under the assumption of a uniform sequence coverage, the expected sequencing depth of the R haplotype is ~ 16 ×, as potato has a tetraploid genome (2n = 4 ×). Paralogous and other repetitive sequences are expected to be present at a multitude of this depth. In order to select for single copy SNPs specific for the resistant haplotype, we decided to keep *k*-mers with a depth from 10 × to 22 ×. These filtered R-bulk specific *k*-mers were further divided into four different sets after comparison with the *k*-mers from the resistant and susceptible parent: inherited from the resistant parent (referred hereafter as “resistance specific *k*-mers”), inherited from the susceptible parent, inherited from both parents or deriving from none of the parents. This last set, probably representing contamination with unrelated biological material, was discarded for subsequent analyses.

In order to select SNPs with high diagnostic value, prior to the development of PCR markers, *k*-mer sets operations were used to eliminate *k*-mers present in different varieties not sharing the same trait. *K*-mers tables were produced for Alegria, Desiree, Kuras and VR808 which are all resistant to the pathotype 1 but susceptible to P2, P6 and P18. The union of the *k*-mers from these varieties was subtracted from the resistance specific *k*-mers set.

For reference genome based downstream analyses, the resistance specific *k*-mers minus S varieties, the R-bulk specific *k*-mers inherited from the susceptible parent and the R-bulk specific *k*-mers inherited from both parents were mapped to identify the genomic region harboring the trait of interest. We used BWA aln (version 0.7.12) [30] to map the R-bulk specific *k*-mers to version v4.03 of the *Solanum phureja* DM1-3 (DM) genome [31, 32]. After mapping, *k*-mers per 1 Mb bins, starting at the beginning of each chromosome, were counted using the bedtools suite (v2.25) [33]. The resulting numbers of *k*-mers per 1 Mb bin were plotted using Microsoft Excel.

For reference genome independent downstream analysis, the paired end reads containing resistance specific *k*-mers were retrieved from the R-bulk and the resistant parent and, successively, assembled de novo. We used an in-house developed script to retrieve the PE reads containing at least one of the resistance specific *k*-mers. The de novo assembly of the extracted reads was done using SPADes (v3.11.1) [34] (command line options: -k 21, 33, 55, 77 –careful –pe1-1 –pe1-2 –pe2-1 –pe2-2). The corresponding scaffolds are mainly derived from the haplotype bearing the resistance and are referred to as the R-haplotype scaffolds. To compare the CoSSA workflows with and without using the reference genome, we mapped the resistance specific *k*-mers to the R-haplotype scaffolds using BWA aln and allowing no mismatches (command line option: -n 0). The *k*-mers mapping to the three biggest R-haplotype scaffolds were extracted using SAMtools view (v1.3) [35] and mapped to the potato reference genome using BWA aln and allowing 0, 1 and 2 mismatches. The number of reads mapping to DM was determined using SAMtools flagstat.

*K*-mer sets algebra was also used for pedigree analyses. To check if different varieties sharing the same trait were identical by descent, the number of *k*-mers shared with the resistance specific *k*-mers were counted. For this purpose, the intersections between the resistance specific *k*-mers not present in the four susceptible varieties and the *k*-mers from the two pedigree clones (Bzura and BRA9089) were made. Successively, the resulting *k*-mers were mapped to the reference genome or to the R-haplotype scaffolds.

### Minimum input required by CoSSA

To assess the influence of the sequencing depth on the CoSSA results, we randomly sampled subsets of reads from the R-bulk, the S-bulk, and the two parents using seqtk (v1.2) to simulate sequencing depths of 10 × and 5 × of the haploid genome. CoSSA was applied to these two datasets, changing only the R-bulk specific *k*-mers depth cut-off which was proportionally adapted to *k*-mers with a depth comprised between 6 × and 14 × and *k*-mers with a depth between 3 × and 7 × for the simulations with a depth of 10 × and 5 × respectively. To assess if we could identify the same loci with the lower depth datasets as with the full dataset, we calculated the signal to noise ratio (SNR) for the 10 × and 5 × simulations, with or without intersections from the parents. Without the parents intersections, the SNR was calculated as being the average number of R-bulk specific *k*-mers mapping under the peak (the peak boundaries are defined as identified with the full dataset) divided by the number of R-bulk specific *k*-mers mapping to the rest of the chromosome. If the parent’s intersections were included, the SNR was calculated as being the ratio between the average of R-bulk specific *k*-mers coming from the specific donor parent under the peak and the average of R-bulk specific *k*-mers coming from the donor parent mapping to the rest of the chromosome.

To test if sequence input for the CoSSA workflows could be minimised further, we subtracted the S-bulk *k*-mers from the resistant parent (Kuba). We kept the *k*-mers from the difference with a depth comprised between 8 × and 18 × and mapped them to the reference genome. The number of mapped *k*-mers per 1 Mb was counted and compared with the CoSSA results obtained with the two bulks and the two parents.

### Design of PCR markers for validation and diagnostic purposes

A haplotype specific SNP without flanking SNPs within 30 bp on each side has a maximum of *k* (=31) resistance specific *k*-mers mapped to it. To select these “isolated haplotype specific SNPs” for the design of markers, we mapped the resistance specific *k*-mers minus the S varieties *k*-mers to the potato reference genome DM (v4.03). To select SNPs coming from Ludmilla, we mapped the R-bulk specific *k*-mers inherited from Ludmilla to the reference genome. From the resulting .bam files, the position of the mapped *k*-mers was inferred using SAMtools and exported to Microsoft Excel. This way, we could pinpoint isolated haplotype specific SNPs because 31 *k*-mers were mapped in a row and because there were no other *k*-mers mapped to the flanking 61 bp sequences. The candidate SNPs were visualized in the .bam file using the Integrative Genomics Viewer (IGV, v2.3.72) (Additional file 3).

To identify resistant haplotype specific SNPs without using the reference genome, we mapped the resistance specific *k*-mers minus the S varieties *k*-mers to the de novo assembled R-haplotype scaffolds and isolated SNPs were pinpointed as described previously. As we mapped the resistance specific *k*-mers without the S varieties *k*-mers to scaffolds belonging to the resistant haplotype, the susceptible variant(s) of the SNPs were not known. Knowing the susceptible variant(s) of the identified SNPs is necessary to design KASP markers. Therefore, we also mapped the R-bulk reads, which contain reads from the resistant haplotype and the seven possible susceptible haplotypes, to the de novo assembly using BWA mem (version 0.7.17). Duplicates were removed using sambamba (v0.5.1) [36]. The susceptible variant(s) of each selected SNP could be visualized in IGV.

### KASP markers genotyping

A first set of Kompetitive Allele Specific Polymorphisms (KASP) markers (Additional file 4), that were used to validate the different CoSSA peaks, was designed by LGC Genomics (LGC, Hoddeston, UK) and tested in a subset (n = 83) of the K* L population. The clones from this subset were selected for historical reasons (first clones of the K*L population). The KASP markers name reflects the physical coordinate of the SNP, relative to DM. Allele specific forward primers and common reverse primers were designed based on the neighboring sequences of the 31 *k*-mers from CoSSA mapped to the reference genome. One KASP marker (chr11_1259552) flanking the resistance locus and two new KASP markers designed for further fine-mapping (chr11_1519485 and chr11_1666090; Additional file 4) were used to genotype the full population (n = 328). Two haplotype specific markers (chr11_1259552, chr11_1772869; Additional file 4) flanking the resistance locus were used to genotype an independent panel of 56 susceptible and resistant potato varieties. The KASP genotyping assays were performed by C. Meijer BV (Rilland, The Netherlands) according to the manufacturer’s instructions (LGC Genomics). In brief, 1.5 µL DNA of a concentration of 5–50 ng/µL in a 1536-well plate was dried in a fan oven for 1 h at 55°C and 1 µL of the PCR mix was added (containing the 2 × KASP Master mix, the primer mix and the water). The PCR was performed in a Hydrocycler 16 and the data reading was performed on a BMG PHERAstar^®^

A second set of three KASP markers (NODE1_7193, NODE2_18410, NODE3_17594; Additional file 4) that were used to validate the “CoSSA without reference genome” workflow was designed in house using Primer3 [37]. The three markers were designed for SNPs from the three longest R-haplotype scaffolds and tested in the subset of 83 K*L progeny clones. These KASP assays were performed according to the manufacturer’s guidelines (LGC Genomics, Hoddesdon, UK) in a total volume of 10 µL with 5 to 50 ng of genomic DNA on a BioRad CFX96^TM^ Real-Time System machine.

Chi^2^ tests were used to test the goodness of fit of the segregation of the tested markers with the expected 1:1 segregation ratio. Kruskal–Wallis tests were performed using R v3.2.3 to validate the association of the tested markers with wart disease resistance.

## Results

### Distribution of resistance in the K*L population

The resistance of the entire full-sib population (n = 328) and the two parents to pathotypes 2, 6 and 18 was assessed (Additional file 1). The distribution of resistance to the pathotypes 2 and 6 segregated in a bimodal fashion in a 1:1 ratio (χ^2^ test *p*-value > 0.05; Additional file 5 A, B, E). The pathotype 18 scores were skewed towards susceptibility (χ^2^ test *p*-value < 0.001; Additional file 5C) which can be explained by a weaker resistance of Kuba to pathotype 18. Nevertheless, the Pearson correlations between the resistance scores for the three pathotypes were very high (Additional file 5D): 0.93 between P2 and P6, 0.91 between P6 and P18 and 0.89 between P2 and P18. These observations suggested the presence of a single major resistance locus for all three pathotypes and possibly a minor effect locus improving resistance to pathotype 18. When transforming the quantitative scores into a qualitative trait (Resistant or Susceptible, Additional file 1), we observed a perfect co-segregation between the three pathotypes. Only a few exceptions were found where the phenotypic classification for resistance against P2, P6 and P18 did not co-segregate. Most of these exceptions might be explained by the choice of the threshold to call a clone resistant or susceptible.

### Design of a CoSSA workflows for gene mapping

To identify sequence variants linked to the wart disease resistance from the K*L population, we pursued a Bulked Segregant Analysis combined with Whole Genome Sequencing. We selected 17 fully resistant and 17 fully susceptible plants to the three pathotypes to compose a resistant bulk (R-Bulk) and a susceptible bulk (S-Bulk), respectively. Selected clones are highlighted in Additional file 1. The R-bulk, the S-bulk, Kuba (R parent) and Ludmilla (S parent) were sequenced with Illumina 151 bp PE reads. The calculated sequencing depth obtained for each sample was of 62 ×, 82 ×, 72 × and 70 ×, respectively, assuming an 840 Mb genome (Additional file 6). It was decided not to use complete reads but subsequences (*k*-mers) of 31 nucleotides instead. Indeed, sequences with an invariable length can be unambiguously compared using set algebra. These basic set operations are less computationally demanding than methods requiring reads alignment to a reference and allow to work independently from a reference. *K*-mer tables (*k* = 31), containing the unique *k*-mers and their frequency, for the four samples were produced and the *k*-mers with a depth of one were removed (Additional file 6). For Kuba and Ludmilla, we observed peaks of *k*-mers at four different *k*-mer frequencies. They corresponded to sequencing errors and PCR duplicates (low depth), the *k*-mers present in one haplotype (simplex; 13 for both Kuba and Ludmilla), two haplotypes (duplex), and the *k*-mers present in three haplotypes (triplex) or four haplotypes (quadruplex) (Additional file 7). The *k*-mers with a depth > 52 (4× 13 ×) were assumed to be originating from multi-copy sequences. Except for the first two peaks (errors and single copy), no distinct frequency peaks could be observed in the bulks. Most likely, the remaining peaks are diffuse because the bulk samples were composed of 17 different DNA samples from individuals containing genetic variants from eight haplotypes. From these eight haplotypes, only the resistant haplotype was present in each R-bulk individual which, theoretically, would be twice the frequency of the single copy peak (2 * 8 = 16 for the R haplotype).

In order to select *k*-mers linked to resistance, we subtracted the S-bulk *k*-mers (Nu (number of unique *k*-mers) = 1,669,156,268; Nt (total number of *k*-mers) = 45,467,875,801) from the R-bulk *k*-mers set (Nu = 1,601,951,563; Nt = 34,475,435,516) to obtain the R-bulk specific *k*-mers (Nu = 88,774,024; Nt = 260,595,070) (Additional file 6). After enriching for simplex *k*-mers using a *k*-mer depth threshold between 10 < Nt *k*-mers < 23, we retained 1,979,520 unique *k*-mers (Nt = 23,219,641). These unique *k*-mers were then divided into four different subgroups: 624,608 unique *k*-mers inherited from Kuba (resistance specific *k*-mers), 197,014 unique *k*-mers inherited from Ludmilla, 329,529 unique *k*-mers inherited from both parents and 828,369 unique *k*-mers inherited from none of the parents. From this last set of *k*-mers, 69% could be mapped to the potato reference genome. This set was not used for further analysis because they were likely due to sequencing errors and DNA contamination from an unrelated potato genotype.

**Fig. 2:**
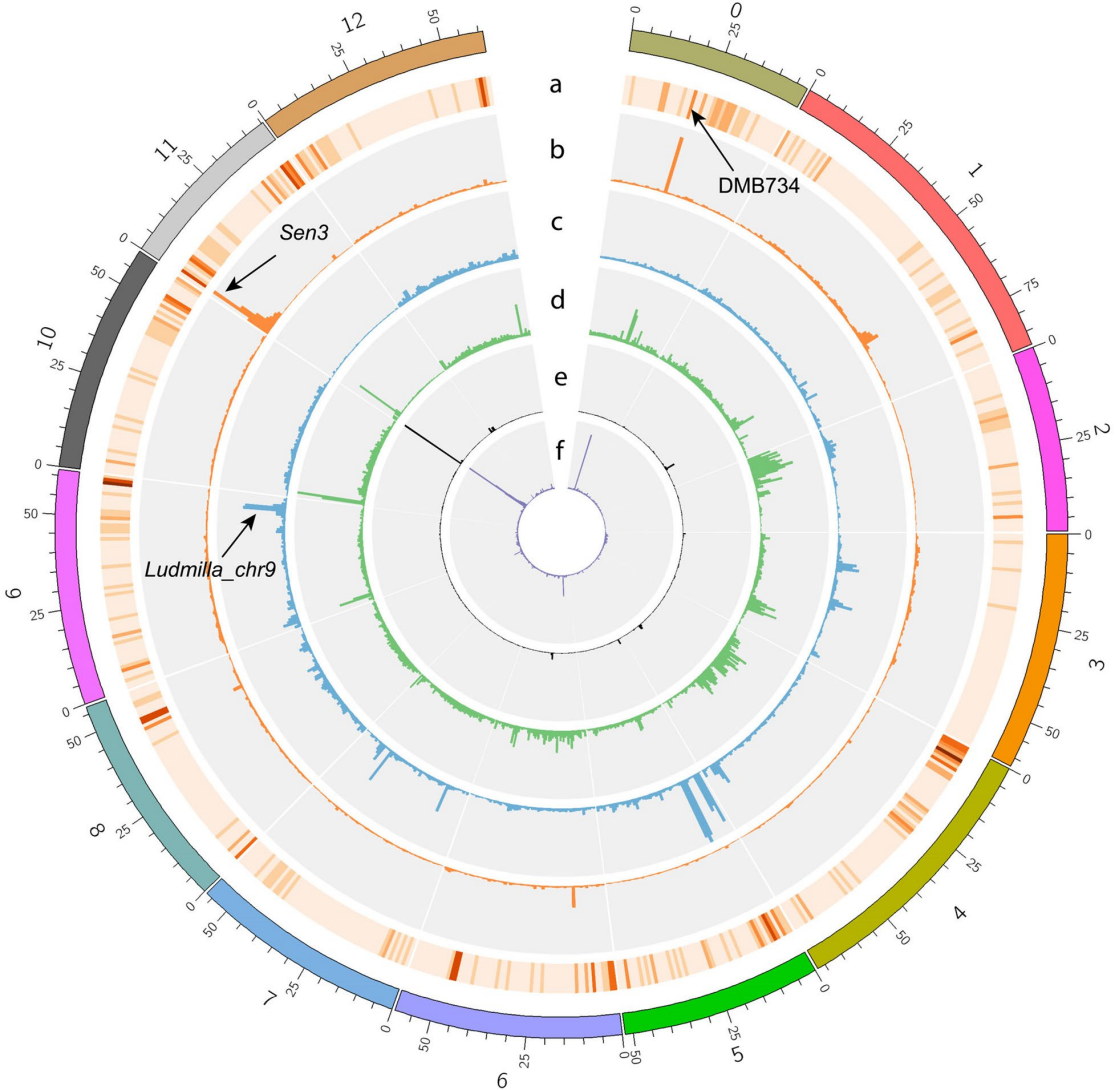
Comparative subsequence sets analysis in the K*L population. The R-bulk specific *k*-mers from three different sets are mapped to the 12 chromosomes of DM and to the unanchored scaffolds (chromosome 0, v4.03). For each chromosome, the average of the number of R-bulk specific *k*-mers mapped has been calculated and for each 1 Mb bin, the number of *k*-mers mapped from each set has been transformed to a percentage of the chromosome average. **a** Number of NLR genes per bin (1 Mb) from the DM genome according to [7]. **b** R-bulk specific *k*-mers minus *k*-mers present in the susceptible varieties inherited from Kuba (y_max_ = 1208%), **c** the *k*-mers inherited from Ludmilla (y_max _= 469%), **d** the *k*-mers inherited from both parents (y_max _= 516%), **e** intersection of the *k*-mers from **b** with the *k*-mers from BRA9089 (y_max _= 428%), **f** intersection of the *k*-mers from **b** with the *k*-mers from Bzura (y_max _= 1205%). The arrows indicate the validated resistance loci and the position of the DMB734 scaffold

To further increase the haplotype specificity of the resistance specific *k*-mers, we removed from the resistance specific *k*-mers the *k*-mers that were also found in the susceptible varieties Alegria, Desiree, Kuras and VR808 (S varieties). The sequencing depth obtained respectively for each tetraploid variety was 46 ×, 46 ×, 43 × and 45 × (Additional file 6). From 624,608 resistance specific *k*-mers, 319,443 *k*-mers remained after subtracting the S varieties *k*-mers (Additional files 6 and 8). The unique resistance specific *k*-mers minus the S varieties and the unique R-bulk specific *k*-mers inherited from Ludmilla and from both parents (845,986 unique *k*-mers in total) were mapped to 1 Mb bins of the potato reference genome DM (Fig. 2, Additional file 8). The highest *k*-mers peak was identified on the first 5 Mb of chromosome 11, where 13% of all the R-bulk specific *k*-mers sets mapped. From these *k*-mers, 84.3% were inherited from Kuba, the resistant parent (resistance specific *k*-mers minus S varieties). Interestingly, we observed another peak of resistance specific *k*-mers mapping to the 20–21 Mb bin of the chromosome 0, and specifically to the unanchored superscaffold PGSC0003DMB000000734 (referred hereafter as DMB734). Several smaller peaks of *k*-mers could be observed in other genomic regions (Fig. 2). The larger and smaller *k*-mers peaks might represent major and minor effect loci, a hypothesis that required validation. We decided to design markers to validate the two major peaks and five minor peaks of R-bulk specific *k*-mers coming from Ludmilla: on chromosome 3 (14–17 Mb; 0.39% of the mapped 3 R-bulk specific *k*-mers sets), chromosome 4 (64–68 Mb; 0.61%), chromosome 5 (0–3 Mb; 1.46%; 7–10 Mb; 2.1%), and chromosome 9 (55–57 Mb; 0.62%) (Additional file 8).

### Validation of the CoSSA results

To validate the hypothesised resistance loci positions as identified with CoSSA, we set out to design haplotype specific KASP markers for the two larger and five of the smaller *k*-mers peaks identified. To increase the chances to select SNPs with diagnostic value, we removed from the resistance specific *k*-mers the *k*-mers that were also found in the susceptible varieties Alegria, Desiree, Kuras and VR808. When comparing the peak of resistance specific *k*-mers mapping to chromosome 11 before and after the subtraction of the S varieties *k*-mers (Additional file 8F), we observed that the highest peak shifted from the 3–4 Mb bin to the 1–2 Mb bin. This suggested that a high proportion of frequently occurring SNPs from multiple ancestral origin mapped to the 3–4 Mb bin.

From these *k*-mer sets, “isolated SNPs” were selected to design 24 KASP markers, covering all 7 CoSSA peaks, which were used to genotype a subset of 83 progeny clones from the K*L population. Chi^2^ tests were used to validate the expected 1:1 segregation pattern of the markers (Table 1). All the markers followed the expected ratio except chr11_5505183 for which the segregation was skewed toward the alternative allele (Table 1; Additional file 1). The KASP markers from chromosome 11 and DMB734 largely co-segregated and were strongly (*p*-values < 0.001) associated with pathotypes 2, 6 and 18 resistance as determined using Kruskal Wallis tests. Upon construction of a linkage map, it was found that DMB734 markers were positioned between markers corresponding to 1,259,552 bp and 1,772,869 bp of chromosome 11. So most likely, DMB734 must be positioned inside one of the two gaps between DMB148 and DMB505. Despite this misassembly, the order of the chromosome 11 markers and the order of the markers on DMB734 were conserved within the *Sen3* locus in comparison with the reference genome.

**Table 1:** Validation of the CoSSA SNPs

SNP	R allele donor	Association with P2 resistance*	Association with P6 resistance*	Association with P18 resistance*	χ^2^	χ^2^ test significance
chr00_20792298	Kuba	2.1E−14	1.2E−14	8.3E−15	1.5	ns
chr00_20801212	Kuba	1.3E−14	7.3E−15	4.8E−15	1.2	ns
chr00_20858042	Kuba	2.1E−14	1.2E−14	8.3E−15	1.5	ns
chr00_20872536	Kuba	1.3E−14	7.3E−15	4.8E−15	1.2	ns
chr03_15683229	Ludmilla	6.8E−01	6.9E−01	3.0E−01	0.1	ns
chr04_64488861	Ludmilla	6.6E−01	9.7E−01	3.8E−01	0.1	ns
chr04_65204393	Ludmilla	4.5E−01	1.1E−01	2.0E−01	1.5	ns
chr05_2117034	Ludmilla	3.8E−01	9.4E−02	1.4E−01	0.0	ns
chr05_8795719	Ludmilla	2.0E−01	9.0E−02	1.6E−01	0.0	ns
chr05_9254557	Ludmilla	2.3E−01	8.4E−02	1.8E−01	0.0	ns
chr09_55113777	Ludmilla	2.4E−03	9.0E−04	4.2E−03	0.0	ns
chr09_55354021	Ludmilla	4.6E−03	1.3E−03	5.9E−03	0.1	ns
chr11_799292	Kuba	7.3E−13	6.7E−14	1.5E−13	0.4	ns
chr11_1259552	Kuba	2.0E−12	1.8E−13	4.5E−13	0.8	ns
chr11_1680269	Kuba	2.2E−14	1.2E−14	7.7E−15	1.5	ns
chr11_1772869	Kuba	2.9E−13	1.8E−13	1.4E−13	0.6	ns
chr11_1919971	Kuba	2.2E−13	1.4E−13	1.1E−13	0.8	ns
chr11_2040481	Kuba	2.2E−13	1.4E−13	1.1E−13	0.8	ns
chr11_2298154	Kuba	2.1E−12	9.7E−12	1.1E−12	1.8	ns
chr11_2687396	Kuba	3.6E−11	6.0E−11	1.9E−11	2.1	ns
chr11_2815640	Kuba	2.5E−11	5.5E−11	1.4E−11	1.5	ns
chr11_3325292	Kuba	1.2E−09	4.6E−09	7.7E−10	2.1	ns
chr11_4362386	Kuba	1.0E−09	3.0E−08	3.4E−09	1.0	ns
chr11_5505183	Kuba	4.8E−06	6.4E−04	4.3E−04	6.5	< 0.01

χ^2^ tests: null hypothesis: the marker follows a 1:1 segregation pattern

*ns* not significant

*p-values of association between KASP marker results and Glynne-Lemmerzahl tests of 2016 (Kruskal–Wallis tests)

Markers designed in four of the smaller CoSSA peaks were not associated with resistance (*p*-values > 0.05). Only the markers chr09_55113777 and chr09_55354021 were associated with pathotypes 2, 6 and 18 resistance (*p*-values < 0.01; Table 1). This second resistance locus was inherited from the susceptible parent Ludmilla and shows a smaller effect on resistance than the chromosome 11 locus (Additional file 9). This shows that CoSSA can identify major and minor loci, but also false positives. Hence, the SNPs identified in the peaks require validation through marker analysis.

### CoSSA data as a source for sequence variants in fine-mapping

**Fig. 3:**
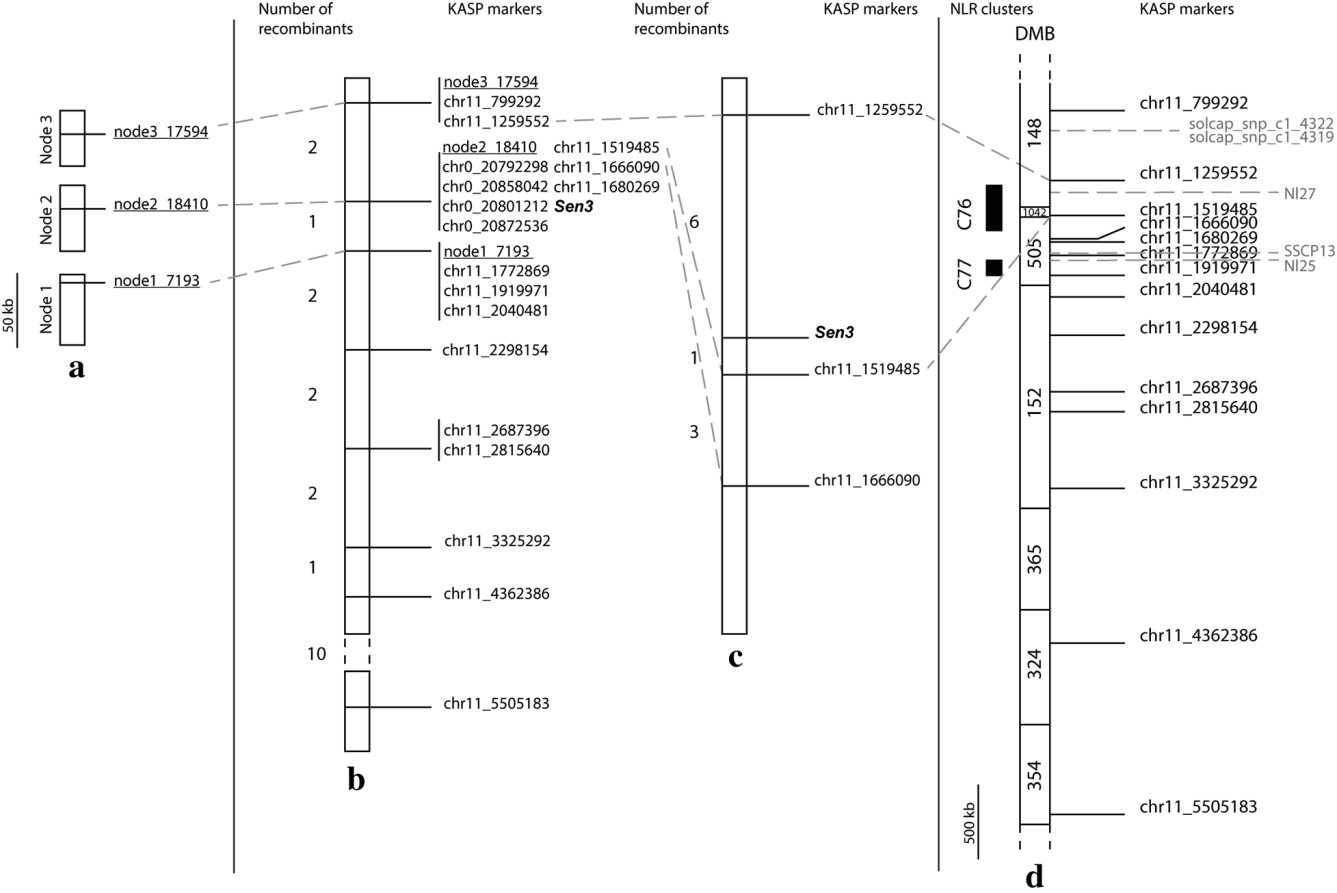
Genetic and physical maps of the resistant haplotype. **a** Physical map of the three longest scaffolds of the R-haplotype de novo assembly. **b** Genetic map of the resistance region in a subset (n = 83) of the Kuba x Ludmilla population. The chromosome 0 scaffold could be anchored to the resistance interval. The analysis of recombinants allowed to fine-map the region flanked by chr11_1259552 and chr11_1772869. The markers developed without using the potato reference genome are underlined. **c** Genetic map of the resistance region in the full Kuba x Ludmilla population (n = 328). The resistance locus was fine-mapped to an interval between chr11_1259552 and chr11_1519485. **d** Physical map of the chromosome 11 first 6 Mb region. The KASP markers developed to screen the Kuba x Ludmilla population are in black on the right of the haplotype and on the left are the NLR clusters [7]. The markers in grey are the markers used to map *Sen1* (Nl25 and Nl27) [16, 18] and to map Karolin’s resistance (solcap markers and SSCP13) [22]

In the subset of 83 progeny clones, we could identify 20 recombinants between markers chr11_799292 and chr11_5505183 on the chromosome 11 haplotype (Fig. 3b; Additional file 1). Three informative recombinants indicated that the locus giving resistance to all the tested pathotypes, which we will refer to as *Sen3*, is within a 513,317 bp window relative to the reference genome (excluding DMB734; flanked by chr11_1259552 and chr11_1772869). We used the CoSSA resistance specific *k*-mers without the S varieties *k*-mers to design two more KASP markers inside the resistance interval (chr11_1519485 and chr11_1666090). Screening of the full K*L population (n = 328) with chr11_1259552, chr11_1519485 and chr11_1666090 narrowed *Sen3* to a 260 kb region between PGSC coordinates 1,259,552 bp and 1,519,485 bp (Fig. 3c). Interestingly, this genomic position contains many TNL (TIR Nucleotide-binding site Leucine-rich repeat) sequences (Fig. 3d) and matched the C76 [7] cluster which is synthenous to the XIa-TNL cluster [38] and RH11.1a clusters [39].

Only two false positives (K*L9006 and K*L9201) and one false negative (K*L9128) were observed in the full population (n = 328). Because of the scarcity of these observations, we can reasonably assume that they were due to mismatches between the samples used for the phenotyping and genotyping. None of the clones which showed a non-perfect co-segregation of the qualitative resistance traits to P2, P6 and P18 showed a recombination event between chr11_1259552 and chr11_1519485 (Additional file 1).

### CoSSA based gene mapping without a reference genome

Identification of sequence variants by mapping reads to a reference genome has its limitations. The genes or regions of interest may be absent or overly different from the reference. Especially *R* gene clusters are difficult to assemble (as evident from the unanchored DMB734 contig; this study), and divergent, even among closely related haplotypes. We were interested to test if CoSSA could also be used without a reference genome. We extracted 408,281 and 469,251 read pairs from the R-bulk and from Kuba, respectively, which contained at least one of the resistance specific *k*-mers and assembled them. The obtained de novo assembly was composed of 21,306 scaffolds (N_50_ = 1115) ranging in size from 100 until 46,690 bp (Additional file 10). To locate the R haplotype specific variants, we mapped the resistance specific *k*-mers minus the S varieties *k*-mers to the de novo scaffolds. “Isolated SNPs” were selected to avoid KASP assay failure due to (unknown) flanking variants. To know the susceptible allele(s) of each DNA sequence variant, we also mapped the R-bulk reads to the de novo scaffolds. Next, we designed one KASP marker on each of the three longest scaffolds (node1 = 46,690 kb, node2 = 44,756 kb, and node3 = 38,969 kb; Fig. 3a) and tested them in the subset of 83 offspring. The three markers were indeed linked to resistance and could be placed in the linkage map (Fig. 3b). The three markers were specific to the resistant haplotype and the markers present on node3 and node1 were flanking the resistance gene, while the node2 marker fully co-segregated.

To further inquire the benefit of the CoSSA workflow without a reference genome, we mapped the resistance specific *k*-mers to the three longest R-haplotype scaffolds (node1, node2 and node3) allowing no mismatches. The mapped *k*-mers were extracted and mapped to the potato reference genome allowing 0, 1 and 2 mismatches (Additional file 11). When no mismatches were allowed, only 2.95%, 0.58% and 41.85% of the *k*-mers mapping to the nodes 1, 2 and 3 respectively could map to the reference genome. When 1 mismatch was allowed (the mismatch corresponds to the resistance specific SNP present in each *k*-mer), 25.6%, 33.11% and 66.63% of the *k*-mers mapping to nodes 1, 2 and 3 respectively mapped to the reference genome. We released even more the mapping stringency by allowing 2 mismatches during the mapping, which allowed *k*-mers containing 2 SNPs to be mapped. With these settings, 45.17%, 60.78% and 81.85% of the *k*-mers mapping to nodes 1, 2 and 3 mapped to the reference. Overall, the nodes 1 and 2 R-haplotype scaffolds contained only a small portion of *k*-mers that mapped to the reference genome, which can be explained by poor assembly of the reference genome or by huge differences between the DM haplotype compared to the *Sen3* haplotype. The node3 scaffold contained more *k*-mers, which could map to DM. According to our genetic map (Fig. 3b), the DM assembly deviates strongly from the *Sen3* haplotype in the region between the flanking markers, which corresponds to the *R* genes clusters. These results clearly show the advantage of working with a reference genome independent approach.

### CoSSA-based pedigree analysis

To assess which clone in the pedigree of Kuba has been the donor of resistance (Additional file 12), we sequenced BRA9089 (suggested great, great grandparent of Kuba) and Bzura (grandparent of Kuba) and obtained sequencing depths of 51 × and 47 × respectively. We used the resistance specific *k*-mers without the S varieties *k*-mers and made the intersection with Bzura and BRA9089 *k*-mers sets. Bzura and BRA9089 shared respectively 75.7% and 6.8% of the resistance specific *k*-mers without the S varieties *k*-mers. These shared *k*-mers were mapped to the reference genome (Fig. 2). Along the seven first bins of chromosome 11 (Additional file 13A), Bzura shared 99.44% of the *k*-mers with Kuba whereas BRA9089 shared only 12.9% of the *k*-mers with Kuba. These results showed that Bzura shared the full resistant haplotype with Kuba but it was not the case for BRA9089. In order to rule out that crossover events on both sides of the causal gene had resulted in lack of similarity in the *k*-mers lists of Kuba and BRA9089, we focussed on the fine-mapped interval (1,259,552–1,519,485 bp). BRA9089 shared 1.7% of the Kuba resistance specific *k*-mers whereas Bzura hold 99.4% of them. In the de novo assembled R-haplotype scaffolds we described previously, on scaffolds node2 and node1 containing the markers flanking *Sen3*, Bzura shared 99.9% of the R-bulk specific *k*-mers with Kuba and BRA9089 0.02% only (Additional file 13B). These observations strongly suggested that the BRA9089 clone we sequenced was not the donor of resistance.^1^

### Robustness of CoSSA based gene mapping

In the CoSSA workflows, we applied a depth cut-off on the R-bulk specific *k*-mers (10 to 22 ×). We wanted to assess how these upper and lower depth cut-offs affected the CoSSA peaks identification. If the parents were sequenced and no lower cut-off was applied on the R-bulk specific *k*-mers (2–22 ×; Additional file 14A), the signal-to-noise (SNR) ratio of *Sen3* (chromosome 11: 0–5 Mb) was drastically reduced from 45.15 to 6.04 (Additional file 14D). The signal from the peak was still 6 times higher than the noise from the rest of the chromosome so the peak was still identifiable. In the case of the minor effect locus from Ludmilla on chromosome 9 (55–57 Mb), that we will refer as *Ludmilla_chr9*, however, the SNR was reduced from 10.04 to 1.75 which was too low to identify the peak. Therefore, the lower depth cut-off was not necessary to identify the major effect locus but necessary to identify the minor effect one. When no upper cut-off was applied (Additional file 14C), the SNR of both the major effect and the minor effect loci were unaffected: the SNR of *Sen3* was of 45.56 instead of 45.15 when an upper cut-off was applied and the SNR of *Ludmilla_chr9* was of 10.11 instead of 10.04 (Additional file 14D). Therefore, the upper cut-off threshold does not affect the detection of the peaks but impacts the selection against multi copy SNPs.

To determine the minimum input to supply for CoSSA to be efficient, we analysed the CoSSA results when the parents’ sequences were not included in the workflow. When sequencing only the two bulks, the SNR of the *Sen3* peak was halved (45.15 to 22.19; Additional file 14D) but it was still sufficient to clearly identify the peak. For the minor effect locus, however, the SNR was reduced from 10.04 to 1.88 when the parents were not sequenced which was too low to identify the peak. Therefore, sequencing only the two bulks was sufficient to identify the major effect locus but not to identify the minor effect one. When the parents were not sequenced, the *k*-mers lower depth cut-off became vital, as the SNR of *Sen3* was reduced from 22.19 (10 to 22 ×) to 1.38 (2 to 22 ×) which made the peak not identifiable. Moreover, new peaks arose in other positions of the genome (Additional file 14A). These peaks were composed of *k*-mers that were not inherited from either of the parents, likely due to DNA contamination from unrelated biological material as discussed previously. This contamination would not have been removed if the parents had not been sequenced, making the lower depth cut-off the only way to discard it.

**Fig. 4:**
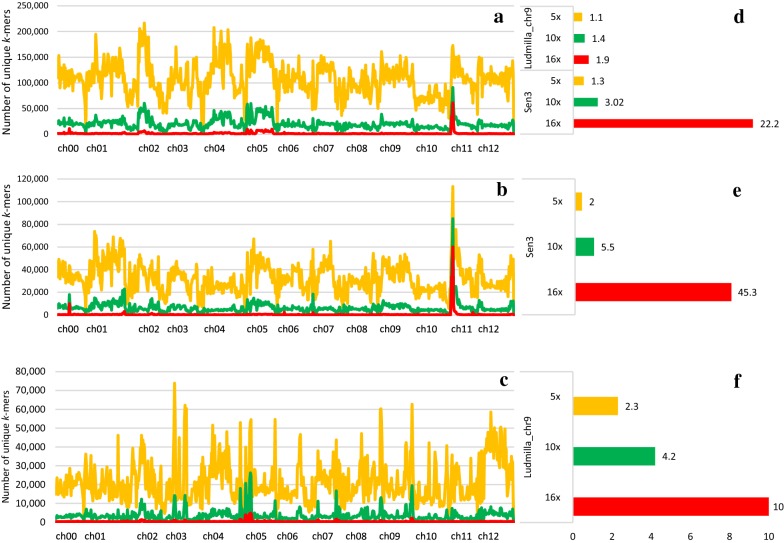
Assessment of the CoSSA minimum required sequencing depth. **a** Total R-bulk specific *k*-mers, **b** R-bulk specific *k*-mers inherited from Kuba (resistance specific *k*-mers) and **c** R-bulk specific *k*-mers inherited from Ludmilla mapped to the potato reference genome using the full sequencing depth (~ 16 ×/haploid) (red), a depth of 10 ×/haploid (green) and a depth of 5 ×/haploid (yellow). **d** Signal to noise ratio (SNR) of the different peaks (*Sen3* and *Ludmilla_chr9*) for the different sequencing depths in the case the parents are not sequenced, **e** SNR of *Sen3* for the different sequencing depths when the parents are sequenced and **f** SNR of *Ludmilla_chr9* for the different sequencing depths when the parents are sequenced

To further minimize the sequence data input for CoSSA, we reduced the sequencing depth of the samples (two bulks and parents) to a depth per haploid genome of 10 × and 5 × instead of 16 × with the full dataset used previously (Additional file 15; Fig. 4). The SNR of *Sen3* was reduced from 22.2 (16 ×) to 3.02 (10 ×) and 1.3 (5 ×) if the parental origin of the *k*-mers was not included in the CoSSA workflow, (Fig. 4a, d). Therefore, a minimum sequencing depth of 10 × per haploid genome was sufficient to identify *Sen3* when no parents were sequenced. However, new peaks arose on chromosomes 2, 4 and 5, which were false positives, as they were not identified with a depth of 16 × per haploid genome (Fig. 4a). For the minor effect locus on chromosome 9, 16 × per haploid genome was already not sufficient for its identification when no parents were sequenced. When the parental origin of the *k*-mers was included in the workflow, *Sen3* SNR was reduced from 45.3 (16 ×) to 5.5 (10 ×) and 2 (5 ×) (Fig. 4b, e). Therefore, even a sequencing depth of 5 × per haploid genome was sufficient to identify the major locus, *Sen3,* if the parents were sequenced. However, as observed previously, new peaks arose on other chromosomes but the chromosome 11 peak was still the highest (Fig. 4b). Regarding the minor QTL (*Ludmilla_chr9*), the SNR was reduced from 10 (16 ×) to 4.2 (10 ×) and 2.3 (5 ×) for 10 × and 5 × (Fig. 4c, f). However, many new peaks arose for the 10 × and 5 × datasets (Fig. 4c). The identification of this locus would then require the design of many more markers for each of these peaks to discard false positives.

Finally, to reduce the CoSSA input by sequencing fewer samples, we simulated the situation where only the resistant parent (Kuba) and the susceptible bulk were sequenced. The difference between Kuba’s *k*-mers sets and the S-bulk’s *k*-mers was made. The *k*-mers with a depth comprised between 8 and 18 ×, according to the peak (n = 13) of simplex *k*-mers in Kuba (Additional file 7), were mapped to the reference genome (Additional file 16). With this reduced input, CoSSA was successful to identify *Sen3*, which had an SNR of 7.39. Obviously, the minor QTL *Ludmilla_chr9* was not found because it is absent from Kuba. However, one smaller peak arose on chromosome 2 (0–13 Mb) with an SNR of 4.28. This peak might be an additional minor effect locus. Again, a validation would be required to rule-out the fact that it is a false positive peak.

### Diagnostic value of the haplotype specific markers for breeding

To assess the diagnostic value of the resistant haplotype specific KASP markers to detect *Sen3* in breeding clones and varieties, we genotyped an independent panel of 56 tetraploid resistant and susceptible varieties with the flanking markers chr11_1259552 and chr11_1772869 (Table 2). The resistant allele of the markers was absent in the tested varieties susceptible to the pathotypes 2, 6 and 18, including the BRA9089 clone which we showed to lack the *Sen3* haplotype using CoSSA. In 17 of the tested varieties, both flanking markers scored positive. From these varieties, 14 (including Bzura that contained *Sen3* as determined using CoSSA) are known to be fully resistant to pathotypes 1, 2, 6 and 18. For the other three varieties (Gawin, Rudawa and Sonda), only a partial pathotype resistance pattern was known. We observed several false negative varieties in the dataset, which were resistant to the pathotypes 2, 6 and/or 18 but did not score positive to the tested markers. These varieties likely got their resistance from another source than *Sen3*.

**Table 2:** Sen3 markers validation in a panel of 56 varieties

Variety	Registration		Flanking makers		Resistant to				Clone in pedigree		
	Country	Year	FM1	FM2	P1	P2	P6	P18	Ora	BRA-9089	Capella
Actaro	HOL	2013	0	0	R	R	R	R			
Adam	POL	2005	1	1	R	R	R	R	?	?	?
Alegria	BRD	2003	0	0	S	S	S	S			
AM78-3704	HOL		0	0	R	R	R	R			
Andante	BRD	2003	0	0	R	R	R	R			
Antares	DDR	1961	0	0	R	R		R			
Apollo	DDR	1956	0	0	R	R		R			
Aventra	HOL	2005	0	0	S	R	R	R			
Axion	HOL	2008	0	0	R	R	R	R			
Belita	HOL	1986	0	0	R	R	R	R			
Bintje	HOL	1910	0	0	S	S	S	S			
BRA9089	GER		0	0	S	S	S	S			
Bzura	POL	1983	1	1	R	R	R	R	y	y	y
Capella	GER	1943	0	0							
Carrera	HOL	1999	0	0	R	S	R	S			
Cekin	POL	2004	1	1	R	R	R	R	?	?	?
Celandine	HOL	2013	0	0	S	S	R				
Constantina	BRD		1	1	R	R	R	R	?	?	?
Delcora	HOL	1988	0	0	R	R	R	S			
Deodara	GER	1913	0	0	S	S	S	S			
Desiree	HOL	1962	0	0	R	S	S	S			
Eurotonda	BRD		1	1	R	R	R	R	?	?	?
Euroviva	BRD		0	0	R	R	R	R			
Gandawa	POL	2004	1	1	R	R	R	R	?	?	?
Gawin	POL	2010	1	1	R			R	?	?	?
Giewont	POL	1955	0	0	R	S	S				
Ibis	POL	1987	1	1	R	R	R	R	n	y	y
Ikar	POL	1996	2	2	R	R	R	R	n	y	y
Karlena	DDR	1988	0	0	R	S	S	S			
Karolin	BRD	1990	1	1	R	R	R	R	y	y	y
Kuba	POL	1999	1	1	R	R	R	R	y	y	y
Kuras	HOL	1996	0	0	R	S	S	S			
Lorch	SU	1931	0	0	S						
Ludmilla	BRD	2008	0	0	R	S	S	S			
Miriam	BRD	1988	0	0	R	S	S	S			
MPI44-1016-10	GER		1	0	R	S	S	S	n	n	n
MPI44-1016-24	GER		0	0	R	R	R	R			
Monalisa	HOL	1982	0	0	R						
Ora	DDR	1952	1	1	R	R	R	R	–	y	y
Otolia	BRD		0	0	R	S	R	R			
Parella	HOL		0	0	R	R	R	S			
Polessky Rosovy	SU	1978	0	0							
Producent	HOL	1984	0	0	R	S	S	S			
Rudawa	BRD	2007	1	2	R	S	R	S	n	y	y
Saphir	BRD	1960	0	0	R	R	R	R			
Smart	HOL	2008	0	0	R	S	S	R			
Sonda	POL		1	1	R	R	S	S	?	?	?
Spectra	HOL	2016	0	0	R		R				
Talent	BRD	2006	0	0	R	R	R	S			
Tivoli	DEN	2002	0	0	R	R	R	R			
Tomensa	BRD	1989	0	0	S	S	S	S			
Transit	BRD	2009	1	2	R	R	R	R	y	y	y
Ulme	BRD	1991	1	1	R	R	R	R	y	y	y
Ultra	HOL	1999	0	0	S		R				
VR808	HOL	2009	0	0	R	S	S	S			
Zagloba	GER		1	1	R	R	R	R	y	y	y

Flanking markers of *Sen3:* FM1 = chr11_1259552 and FM2 = chr11_1772869. The dosage of the FM1 and FM2 is given (0: absent, 1: simplex, 2: duplex). Pedigree information is only shown if the variety was positive for the *Sen3* markers

?: No pedigree information is available, n: This clone is not in the pedigree, y: This clone is in the pedigree

## Discussion

In this study, we designed CoSSA workflows for gene mapping and related applications in which sequence variants are deduced directly from subsequences (*k*-mers), without the necessity of variant calling software. CoSSA is a quick and robust method to identify a locus involved in the variation of a particular trait. When the biggest datasets in this study were used, the jobs parallelized and run on only one node of 10.5 Gb memory, the CoSSA workflow using a reference genome took half a day. The reference independent workflow required around 30 h. The read pairs extraction step, currently consuming 23 h CPU, could be improved to be quicker (Additional file 2). In this case study, we mapped a major and a minor locus for resistance against pathotypes 2, 6 and 18 of *Synchytrium endobioticum*, the causal agent of potato wart disease. The CoSSA workflows also enabled to identify the major locus independently from a reference genome. Furthermore, CoSSA enabled to select for haplotype specific SNPs to fine-map the *Sen3* locus corresponding to a 260 kb interval in the reference genome (between 1,259,552 and 1,519,485 bp). The haplotype specific SNPs identified in this way proved to have diagnostic power in potato breeding material and pedigree analysis.

*Synchytrium endobioticum* pathotypes 2, 6 and 18 resistance had previously been identified in a similar region as the *Sen3* locus as identified in this study [21, 22]. In the first study, the authors used 195 SSR markers to genotype the resistant parent Karolin and a susceptible parent which identity was not disclosed, as well as two DNA bulks of a tetraploid offspring segregating for pathotypes 1, 2, 6 and 18 resistance [21]. Furthermore, the 8.3 k SolCAP potato SNP array was used to identify markers significantly associated with resistance. The authors could not validate these markers with resistance in a broader panel of 91 distantly related varieties, probably because none of the markers was in coupling phase with the resistance gene. Such ascertainment bias can be circumvented only with haplotype specific SNPs. The failure to extrapolate SolCap markers associated with resistance from a biparental study to a GWAS panel should be expected because the SNP discovery panel of SolCap SNPs is not resistant to wart disease. In a later study, a dihaploid population was generated from the resistant variety Karolin and screened with the 12.8 k SolCAP potato genotyping array [22]. The authors compensated the ascertainment bias caused by the SolCAP array by developing new haplotype specific markers using the RNAseq data obtained from three resistant descendants. The resistant locus was mapped to a region corresponding to a 777 kb interval of the reference genome (939,581 bp and 1,716,722 bp) which overlaps with the 260 kb interval we identified in this study.

In our study, we included Bzura, the resistant grand-parent of the studied population, and BRA9089, a speculated donor for a resistance locus found in the German and Polish potato breeding material [40–42]. Among the 17 varieties from the tested panel holding the *Sen3* markers, eight were bred in Germany and nine in Poland. Unfortunately, we could only trace back the resistant haplotype in Kuba’s pedigree until Ora, a daughter of BRA9089 and Capella. Neither Capella, nor BRA9089 contained *Sen3.* We re-phenotyped the BRA9089 clone that we sequenced and it turned out to be susceptible to the four pathotypes. The impossibility to trace back further than Ora the haplotype in the pedigree could be due to pedigree errors. Another likely explanation is that the Capella and/or BRA9089 clones referred in literature are different from the clones we call Capella and BRA9089 today. Three out of the varieties that harbour the *Sen3* markers do not contain Ora in their pedigree but do have Capella and BRA9089 (Table 2).

It is tempting to affirm that Kuba and Karolin share their resistance Identity-By-Descent. Unfortunately, insufficient pedigree information is available from Karolin. Kuba was tested with the flanking markers Kc8103 and RK36 and turned out to be positive [22]. We tested Karolin with chr11_1259552 and chr11_1772869 from the current study and it was positive as well. Moreover, the authors tested their markers in a panel of potato varieties, 15 of which were included in our panel as well. Gandawa, Gawin, Ibis, Ikar, Karolin, Kuba, Rudawa and Ulme are positive to our markers as well as the markers developed by [22]. However, the pedigree of these varieties offers many opportunities to conceive alternative routes of transmission of resistance genes from different pedigree branches. Our study enabled to trace back the *Sen3* locus until Ora. However, Ora is not present in the pedigree of all of the above-mentioned positive varieties (Table 2). With the current information, we are not able to call the wart resistances from Karolin and Kuba identical by descent. CoSSA analysis would be suitable to prove sequence identity but genomic reads from Karolin are unfortunately not available. However, based on the markers produced in this study and on the *S. endobioticum* pathotype resistance pattern, the Kuba and Karolin resistances are indistinguishable.

Since its development almost 30 years ago [1, 2], Bulked Segregant Analysis strategies are used as an alternative to linkage map based QTL analysis and to identify new markers in unsaturated regions of genetic maps. BSA strategies have the main advantage of reducing the number of samples to be genotyped. In the case of CoSSA, we showed that the minimum requirement to identify *Sen3* was to sequence two samples with contrasting phenotypes (R-bulk and S-bulk) with a sequencing depth for each pool of 10 × per haploid genome (i.e. 40 × for a tetraploid genome), reducing radically the genotyping costs. Indeed, we observed that even in the case of a relatively high number of *k*-mers which were not inherited from any of the parents (likely due to DNA contamination), applying a filter on the resistant bulk specific *k*-mers depth drastically improved the signal-to-noise ratio and allowed to identify the resistance locus segregating (Additional file 14A). If the two parents are included in the CoSSA workflow, the sequencing depth of the pools can be reduced further to 5 × per haploid genome. Sequencing the two parents along with the bulks allowed to remove the previously described set of *k*-mers inherited from none of the parents. It has the other benefit of enabling the identification of minor effect loci. For instance, the minor effect locus inherited from Ludmilla on chromosome 9 would have not been identified without using the sequences of the two parents in the pipeline. Indeed, the SNR was too low, even with a sequencing depth of 16 × per haploid genome, to be identified if the parents were not included in the analysis. Another alternative in CoSSA is to replace the resistant bulk by the resistant parent. This approach has the limitation that it will not identify minor QTLs from the susceptible parent. An advantage could be that it is more sensitive to minor QTLs from the resistant parent. A chromosome 2 peak (0–13 Mb) was observed at the same position when using the full sequencing dataset (Fig. 2). This peak was ignored initially due to low SNR. In future research, we might be able to validate this hypothesis.

The set algebra applied in the current CoSSA workflow is made possible by the fixed length of the subsequences. Such an approach based on set algebra has been implemented in the NIKS algorithm [43] to identify the causal mutations in populations derived from EMS mutagenized plants. The beginning of the NIKS workflow is similar to CoSSA: pools of mutants and wild types are sequenced, *k*-mer lists are produced for each bulk and *k*-mers with a low frequency are removed. Subsequent steps in the CoSSA workflow are clearly different and serve different goals. NIKS workflow is designed to identify the mutation causal for a specific phenotype that segregates. Therefore, the NIKS workflow is a comparative subsequence sets analysis that identifies a “needle in the k-stack”. The CoSSA workflows presented in our study are designed to identify haplotype specific “k-packs from the k-stack”. Moreover, NIKS workflow is not suited to identify k-stacks linked to dominant genes like *Sen3* because NIKS requires the haplotype of interest to be homozygous. Therefore, comparative subsequence set analysis is a powerful approach to address different genetic questions, but each question will require a different workflow.

Although the set algebra allows quick and unambiguous comparisons between samples, it may be stringent as it discards all the first bulk *k*-mers that occur at least 2 times in the second bulk. Therefore, CoSSA works best for major QTLs that can be phenotyped unambiguously. If the phenotyping is not straightforward, linked *k*-mers will be present both in the susceptible and resistant bulks. The use of the set algebra might also have the consequence of missing minor effect loci that will be present in both bulks. A way to reduce the stringency is to increase the number of individuals that compose the bulks. CoSSA offers another way to reduce stringency by increasing the S-bulk *k*-mers depth cut-off, which would not require a bigger population. Instead of removing from the R-bulk all the *k*-mers that occur at least 2 times in the S-bulk, we could remove only the *k*-mers which occur more than $\frac{x}{n} \times m$ with *x* the depth of the S-bulk, *n* the number of individuals in the S-bulk, and *m* the number of phenotyping mistakes allowed (> 0).

The selection of haplotype specific variants was improved by including four potato varieties susceptible to the wart disease. Including these varieties in the CoSSA workflow halved the number of resistance specific *k*-mers. In other words, without this extra-step, the markers we designed had only 50% of chance to be haplotype specific. Moreover, we showed that the peak shifted from 3–4 Mb to 1–2 Mb when removing common *k*-mers present in the four susceptible varieties. This suggested that the 1–2 Mb region contained SNPs with a higher haplotype specificity than the 3–4 Mb region. This might be due to a recombination of the original *Sen3* haplotype, which replaced the centromeric part with a more common haplotype. Therefore, after the removal of the susceptible varieties *k*-mers, only the *k*-mers derived from the *Sen3* introgression remained. We improved the SNPs selection as well by applying a depth cut-off on the resistance specific *k*-mers. The lower depth cut-off allowed to remove noise coming from *k*-mers that were not linked to resistance but were, by chance, not present in the S-bulk or noise coming from PCR duplicated reads containing sequencing errors. The upper cut-off, on the other hand, allowed to select for SNPs coming from single copy regions. The thresholds can be chosen by estimating the depth of the resistant haplotype according to the sequencing depth of the R-bulk, or by determining the *k*-mers frequency peaks such as in Additional file 7.

The markers specific to the *Sen3* haplotype were tested in an independent panel of 56 tetraploid varieties (Table 2) and their diagnostic value was tested. Indeed, none of the susceptible varieties tested were positive to the two flanking markers. Among the 17 varieties that were positive, 14 are known to be resistant to pathotypes 1, 2, 6 and 18. Gawin, Rudawa and Sonda are only known to be resistant to P1 in addition to P18, P6 and P2 respectively. These incomplete resistance patterns could be explained by the fact that: 1. not all pathotypes were tested, 2. the isolates of the tested pathotypes used for phenotyping were derived from different sources and that their different genetic composition causes they are not recognised by *Sen3*, or 3. thresholds for resistance and susceptibility were not sufficiently uniform. We found in our K*L population plants that harboured both flanking markers but that were only partially resistant to P18. Future analyses will learn what the genetic basis for the partial or complete P18 resistance conferred by *Sen3* is.

The previously discussed strategy to include more varieties without the trait of interest in the CoSSA workflow to remove common SNPs could be expanded to an association mapping type of approach. Instead of applying the method on a segregating population, CoSSA could as well be applied on bulks from a broader panel of genotypes with opposite phenotypes. This strategy would be successful only when the causal genes are identical by descent. A similar method, called AgRenSeq, has recently been developed [13] and proved to be efficient to map *R* genes in *Aegilops tauschii*. The authors performed RenSeq, which requires prior knowledge about Nucleotide binding Leucine rich Repeat (NLR) genes repertoire and also introduces a bias to NLR rich regions of the genome and prevent the identification of other genes (non-NLR). CoSSA with WGS data is an unbiased genome wide approach that involves moderate sequencing costs and computation extensive *k*-mer counting instead of computation intensive reference genome dependent variant calling. An untargeted association mapping method based on the use of *k*-mers (HAWK) has been developed [12]. CoSSA differs from HAWK by its simplicity as it does not require advanced statistical methods or strong bioinformatics skills. Moreover, the last step of the CoSSA workflow consisting of assembling the read pairs containing the resistance specific *k*-mers instead of the *k*-mers themselves as in the HAWK pipeline should give a better quality of the assembly. Indeed, by using the *k*-mers, one loses the advantage of the paired end reads that can help resolving repetitive regions as well as duplicated or regions harbouring low complexity levels. We overcame this drawback by selecting the haplotype specific read pairs using the resistance specific *k*-mers.

The de novo assembly of the reads containing the resistance specific *k*-mers and the mapping of the resistance specific *k*-mers to the de novo scaffolds allows to reduce the number of candidate SNPs for which to design markers. Indeed, mapping-free SNP calling methods have been developed [44–46] and could be used in a BSA setup. However, without an assembly, more SNPs would have to be tested to map the locus. Moreover, having the sequences flanking the candidate variants improves the quality of the markers. Additionally, the de novo assemblies allow identification of candidate genes. For instance, [13] using NLR-parser [47] could search for NLR genes in de novo assemblies. However, in the case of NLR genes, it has been shown that a de novo assembly based on short reads might result in chimeric scaffolds [48]. In the same publication, by using PacBio long reads, the authors successfully assembled a complex resistance gene cluster and cloned the causal resistance gene.

In this paper, we mapped *Sen3* to a region containing the complex *R* gene cluster C76 which harbours 8 TNLs [7]. The first locus involved in potato wart disease resistance identified, *Sen1*, was also mapped to chromosome 11 near the C76 cluster [16] (Fig. 3). *Sen3* could be a different paralog from the same cluster as *Sen1*, but it could also be an allelic variant of *Sen1*. Only fine-mapping and the cloning of these genes will answer this question. The C76 cluster is in fact even more complex as the assembly of the DM genome is incomplete in this region (Fig. 3). By adding contig DMB734, we added 3 TNLs and still at least one additional gap remains. This complexity was also reflected in the de novo assembly we generated from the resistant haplotype specific reads which was very scattered. Thus, we were unsuccessful in assembling the *R* gene cluster using the short reads. However, our analyses showed clearly that our assembly of the R haplotype was more complete than DM. Only 11.9% of the resistance specific *k*-mers that mapped to the three longest scaffolds of the de novo assembly could be mapped back to the reference genome with a perfect match and only 38.2% could be mapped allowing one mismatch. This matched our observations that the chromosome 11 *R* gene cluster but also many other *R* gene regions in DM are incomplete.

## Conclusions

Overall, the CoSSA workflows were successful in mapping the potato wart disease resistance locus segregating in a full sib population of tetraploid non-inbred parents, with or without the use of the potato reference genome. This method is both quick and cheap as the bioinformatics pipeline required approximately only half a day and the price of whole genome sequencing using short reads is still decreasing. CoSSA is highly efficient as well: in the reference-free experiment, we needed to screen only three KASP markers to identify the resistance locus and design two valuable flanking markers. Moreover, we showed that the CoSSA de novo assembly of the R haplotype was more complete than the potato reference genome. The CoSSA workflows offer many possibilities to the user such as the quick mapping of major and minor effect loci, the identification of haplotype specific variants, the de novo assembly of haplotype specific reads and the *in silico* inspection of genotypes sharing the same trait. Finally, the different filters settings along the pipeline allow the user to adapt CoSSA to diverse crops and traits.

## Additional files

**Additional file 1.** Phenotypic and genotypic data of the K*L population. The Excel sheet comprises: the genotypic data for all the tested markers, the number of tubers tested per genotype, the average score of the Glynne-Lemmerzahl assays performed in 2016 and 2017 for the pathotypes 2, 6 and 18, the individuals included in the subset (n = 83) used for the validation of the CoSSA results and the individuals selected for the R-bulk and the S-bulk.

**Additional file 2.** CoSSA computational requirements. Computational requirements for the biggest datasets we used in the CoSSA workflows (sequencing depth of 16 ×/haploid genome). We showed that a depth of 10 ×/haploid genome is sufficient when the parents are not sequenced and 5 ×/haploid genome when parents are sequenced which reduces these computational requirements. The different jobs were executed on the WUR Plant Breeding server. Only one node of 10.5 GB memory was used. When the jobs are parallelized for the different CoSSA samples, the reference genome dependent workflow can be run in half a day. For the reference genome independent CoSSA workflow, we advise to allocate more nodes to the reads extraction and to the de novo assembly steps as they require more time. The most time consuming step at the moment which could be improved is the script used to extract the read pairs containing the *k*-mers.

**Additional file 3.** Identification of isolated haplotype specific SNPs for markers development. To identify a good candidate SNP to design diagnostic markers, we mapped the resistance specific *k*-mers minus S varieties to the reference genome and counted how many *k*-mers map to each chromosome bin. Each resistant haplotype specific SNP can have maximum *k* *k*-mers (in our case, 31 31-mers) mapped to itself. We designed most KASP markers for SNPs which had 31 *k*-mers mapped under the main *k*-mers peaks. Fluorescent dyes: F = FAM; H = HEX.

**Additional file 4.** KASP primers used in the study. For each KASP marker used in the study is given: the marker name (chromosome_position), the experiment in which it was used, the population in which it was used, the two forward primer sequences and the common primer sequence.

**Additional file 5.** Resistance distribution in the K*L population. Resistance distribution in the full-sib population (n = 328) for pathotypes 2 (A), 6 (B) and 18 (C). The phenotyping was performed with the Glynne-Lemmerzahl method in 2016. For each resistance category, the proportion of individuals with the R allele of *Sen3* (black), without the R allele (grey) and the proportion of recombinants (black stripes) is given. In (D) are given the Pearson correlations between the resistance mean scores of P2, P6 and P18. Chi square tests were conducted to validate if the resistance to the three pathotypes segregated in a 1:1 ratio in the population (E).

**Additional file 6.** Summary of the sequencing data used and of the CoSSA *k*-mers subsets. For all the samples sequenced, the number of reads and the sequencing depth is given. The number of unique *k*-mers (Nu) and the number of total *k*-mers (Nt) is given for all the sequenced samples and several subsets obtained after basic set operations for different depth cut-offs.

**Additional file 7.** Distribution of the number of total *k*-mers in function of the *k*-mer depth. The total number of *k*-mers (unique *k*-mers x depth) for each sequencing depth from 2 to 100 are represented in this graph for the 4 different samples (red: Kuba, blue: Ludmilla, green: R-bulk, yellow: S-bulk). For the two varieties samples (Kuba and Ludmilla), the peaks of *k*-mers from simplex, duplex, triplex and quadruplex regions are visible. There is a shift in the simplex peak of the bulks as there are 8 possible haplotypes instead of 4 as in the parental genotypes.

**Additional file 8.** CoSSA results with the reference genome. Number of R-bulk specific *k*-mers (depth 10 to 22) mapping to each 1 Mb bin of (A) chromosome 0, (B) chromosome 3, (C) chromosome 4, (D) chromosome 5, (E) chromosome 9 and (F) chromosome 11 of the potato reference genome DM. Red: *k*-mers inherited from Kuba (resistance specific *k*-mers), yellow: *k*-mers inherited from Kuba minus the *k*-mers present in the susceptible varieties, blue: *k*-mers inherited from Ludmilla, green: *k*-mers inherited from both parents.

**Additional file 9.** Markers effects on pathotypes 2, 6 and 18 resistance. Boxplots of the resistance scores for P2, P6 and P18 in function of the allele (0: S allele, 1: R allele) of the KASP markers chr11_1519485 (A) and chr09_55113777 (B) in the Kuba x Ludmilla population subset (n = 83).

**Additional file 10.** De novo assembly statistics. Statistics of the de novo assembly of the resistant haplotype performed.

**Additional file 11.** Comparison of the CoSSA workflows with/without reference. Number and percentage of the *k*-mers mapping to the three biggest scaffolds of the de novo assembly with 0 mismatches that map to the reference genome (DM) as well. During the mapping process to the reference, 0, 1 and 2 mismatches were allowed.

**Additional file 12.** Pedigree of Kuba. The pedigree tree of Kuba (according to [49]) and the known resistance pattern of its ancestors. BRA9089 is present in the pedigree of several varieties resistant to pathotypes 2, 6 and 18 and is thought to be the ancestral donor of their resistance. BRA9089 is present in the resistant grand-parent pedigree (Bzura) and in the susceptible grand-parent (Karlena) pedigree as well.

**Additional file 13.** Pedigree CoSSA analysis. Number of unique *k*-mers in common between the resistance specific *k*-mers without the S varieties *k*-mers and Bzura (the resistant parent of Kuba), BRA9089 (putative resistant donor) and Kuba, (A) mapped to the reference genome (chromosome 11, 0–7 Mb, DMB734 *k*-mers were added to the 1–2 Mb bin) (B) mapped to the 10 longest scaffolds of the de novo assembly. Orange: Kuba, blue: Bzura, black: BRA9089. The bars numbers represent the percentages of the intersection *k*-mers.

**Additional file 14.** Assessment of the depth cut-offs on the CoSSA results. Assessment of the effect of the lower and upper depth cut-off on the CoSSA output. (A) CoSSA output without the lower depth cut-off (R-bulk specific *k*-mers with a depth from 2 to 22 ×), (B) CoSSA output with the lower and upper cut-offs (R-bulk specific *k*-mers with a depth from 10 to 22 ×), (C) CoSSA output without the upper depth cut-off (R-bulk specific *k*-mers with a depth from 10 to ∞). Red: k-mers inherited from Kuba (resistance specific *k*-mers), blue: *k*-mers inherited from Ludmilla, green: *k*-mers inherited from both parents, grey: *k*-mers inherited from none of the parents. (D) Signal to noise ratio (SNR) for *Sen3* and *Ludmilla_chr9*. Black: SNR for CoSSA results when no lower cut-off is applied (2 to 22 ×), stripped: SNR for CoSSA results when an upper and a lower cut-offs are applied (10 to 22 ×), grey: SNR for CoSSA results when no upper cut-off is applied (22 to ∞).

**Additional file 15.** Description of the datasets used in the CoSSA simulations with lower sequencing depths. Sequencing depth of the full sequencing data (16 ×/haploid genome) and the two randomly sampled subsets to simulate depths of 10 × and 5 × per haploid genome. For each subset, the *k*-mers depth cut-off used in the CoSSA is given.

**Additional file 16.** CoSSA applied with Kuba and the S-bulk only. Kuba specific *k*-mers (depth 8 × to 18 ×) obtained by the difference between Kuba and the S-bulk mapped to the reference genome.

## Data Availability

The datasets used and/or analysed during the current study are available in the published manuscript or available from the corresponding author on reasonable request.

## Footnotes

1 To validate that the clone BRA9089 that we sequenced was indeed not the resistance donor, we decided to re-phenotype it. The phenotyping results showed that our sample of BRA9089 was indeed susceptible to pathotypes 1, 2, 6 and 18 (mean scores of 4.4, 4.2, 5 and 4.8 respectively). We also genotyped BRA9089 with chr11_1259552 and chr11_1772869 which was negative to the tested markers (Table 2).
