# Supplementary material for: Comparative Subsequence Sets Analysis (CoSSA) is a robust approach to identify haplotype specific SNPs; mapping and pedigree analysis of a potato wart disease resistance gene Sen3
Source: Plant Methods. 2019 May 29;15:60. doi: 10.1186/s13007-019-0445-5 (PMC6540404; doi:10.1186/s13007-019-0445-5)
Supplement: Supplementary file 2 — Additional file 2. CoSSA computational requirements. Computational requirements for the biggest datasets we used in the CoSSA workflows (sequencing depth of 16 ×/haploid genome). We showed that a depth of 10 ×/haploid genome is sufficient when the parents are not sequenced and 5 ×/haploid genome when parents are sequenced which reduces these computational requirements. The different jobs were executed on the WUR Plant Breeding server. Only one node of 10.5 GB memory was used. When the jobs are parallelized for the different CoSSA samples, the reference genome dependent workflow can be run in half a day. For the reference genome independent CoSSA workflow, we advise to allocate more nodes to the reads extraction and to the de novo assembly steps as they require more time. The most time consuming step at the moment which could be improved is the script used to extract the read pairs containing the k-mers. [file 13007_2019_445_MOESM2_ESM.docx]

**Additional file 2**

Computational requirements for the biggest datasets we used in the CoSSA workflows (sequencing depth of 16x/haploid genome). We showed that a depth of 10x/haploid genome is sufficient when the parents are not sequenced and 5x/haploid genome when parents are sequenced which reduces these computational requirements. The different jobs were executed on the WUR Plant Breeding server. Only one node of 10.5GB memory was used. When the jobs are parallelized for the different CoSSA samples, the reference genome dependent workflow can be run in half a day. For the reference genome independent CoSSA workflow, we advise to allocate more nodes to the reads extraction and to the *de novo* assembly steps as they require more time. The most time consuming step at the moment which could be improved is the script used to extract the read pairs containing the *k*-mers.

|  | Example dataset | CPU time (hh:mm:ss) | Wallclock Time | Max vmem | Input size | Output size |
| --- | --- | --- | --- | --- | --- | --- |
| Reads trimming using Trimmomatic | S-bulk  228,585,110 read pairs  Depth ~82x | **01:36:07** | **01:27:54** | **2.7 GB** | Forward + Reverse fastq.gz files = **44.3 GB** | **169 GB** |
| *K*-mer analysis using Glistmaker | S-bulk trimmed reads in pair Depth ~82x | **03:34:11** | **01:50:35** | **116.3 GB** | Trimmed reads in pair = **143 GB** | **68.4 GB** |
| Remove *k*-mers with frequency = 1 | S-bulk *k*-mers (union file of Reads1 and Reads2) | **00:08:42** | **00:08:36** | **95.9 GB** | S-bulk *k*-mers list =  **50.3 GB** | **18.8 GB** |
| Subtract S-bulk from R-bulk | R-bulk and S-bulk (from 16x dataset) *k*-mers depth >1 | **00:01:23** | **00:01:07** | **36.6 GB** | R-bulk + S-bulk *k*-mers lists = **38.3 GB** | **1.0 GB** |
| Mapping the resistance specific *k*-mers to DM | Resistance specific *k*-mers (from 16x dataset) | **00:05:41** | **00:05:37** | **1.3 GB** | Resistance specific *k-*mers = **21.3 KB**  Ref genome = **766.3 MB** | **103.3 KB** |
| Extract reads containing resistance specific *k*-mers | Kuba PE reads (~72x) and resistance specific *k*-mers (from 16x dataset) | **23:02:07** | **23:04:15** | **85.3 MB** | Kuba PE reads = **142.4 GB**  Resistance specific *k*-mers = **21.3 KB** | **333.2 KB** |
| *de novo* assembly of the R-bulk and PR extracted PE reads | Kuba and R-bulk extracted PE reads (from 16x dataset) | **04:11:32** | **04:11:44** | **4.3 GB** | Kuba extracted PE reads = **333.2 KB**  R-bulk extracted PE reads = **290 KB** | **22.5 KB** |
