# Supplementary material for: Comparative Subsequence Sets Analysis (CoSSA) is a robust approach to identify haplotype specific SNPs; mapping and pedigree analysis of a potato wart disease resistance gene Sen3
Source: Plant Methods. 2019 May 29;15:60. doi: 10.1186/s13007-019-0445-5 (PMC6540404; doi:10.1186/s13007-019-0445-5)
Supplement: Supplementary file 3 — Additional file 3. Identification of isolated haplotype specific SNPs for markers development. To identify a good candidate SNP to design diagnostic markers, we mapped the resistance specific k-mers minus S varieties to the reference genome and counted how many k-mers map to each chromosome bin. Each resistant haplotype specific SNP can have maximum k k-mers (in our case, 31 31-mers) mapped to itself. We designed most KASP markers for SNPs which had 31 k-mers mapped under the main k-mers peaks. Fluorescent dyes: F = FAM; H = HEX. [file 13007_2019_445_MOESM3_ESM.docx]

**Additional file 3**

To identify a good candidate SNP to design diagnostic markers, we mapped the resistance specific *k*-mers minus S varieties to the reference genome and counted how many *k*-mers map to each chromosome bin. Each resistant haplotype specific SNP can have maximum *k* *k*-mers (in our case, 31 31-mers) mapped to itself. We designed most KASP markers for SNPs which had 31 *k*-mers mapped under the main *k*-mers peaks. Fluorescent dyes: F = FAM; H = HEX.

**
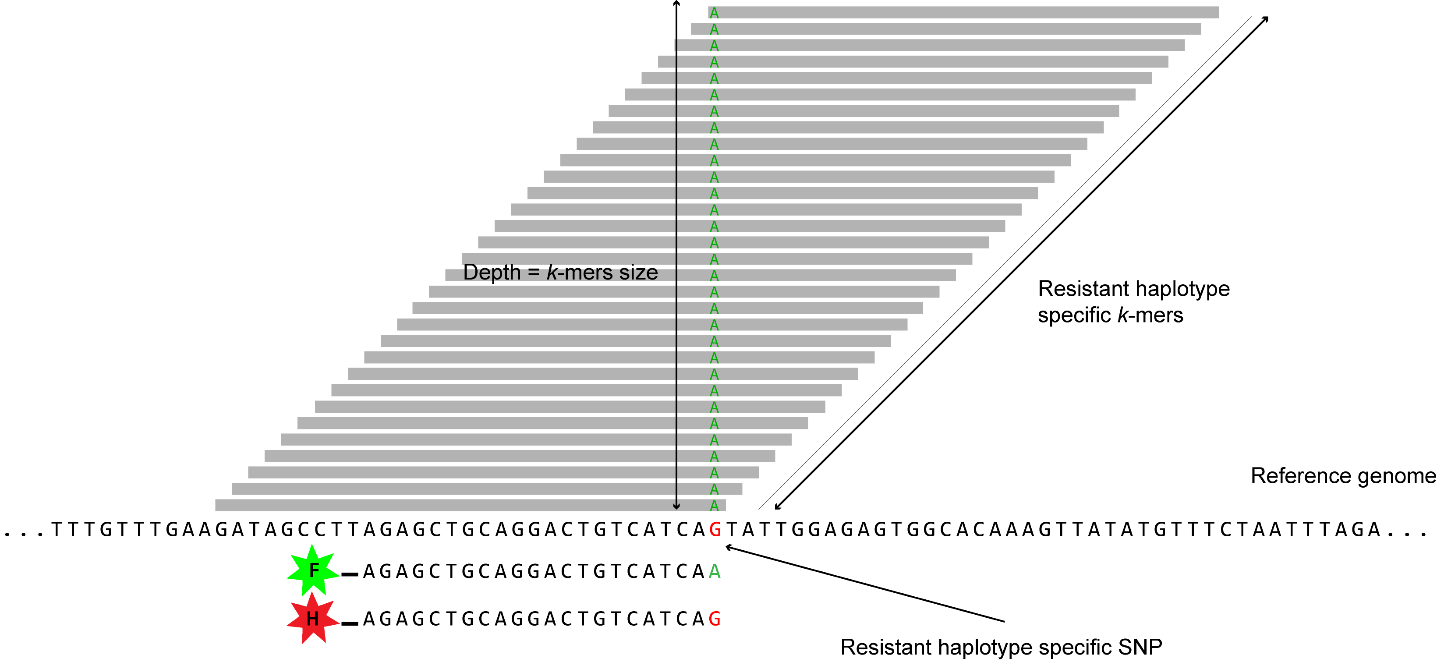
**
