# Supplementary material for: Comparative Subsequence Sets Analysis (CoSSA) is a robust approach to identify haplotype specific SNPs; mapping and pedigree analysis of a potato wart disease resistance gene Sen3
Source: Plant Methods. 2019 May 29;15:60. doi: 10.1186/s13007-019-0445-5 (PMC6540404; doi:10.1186/s13007-019-0445-5)
Supplement: Supplementary file 5 — Additional file 5. Resistance distribution in the K*L population. Resistance distribution in the full-sib population (n = 328) for pathotypes 2 (A), 6 (B) and 18 (C). The phenotyping was performed with the Glynne-Lemmerzahl method in 2016. For each resistance category, the proportion of individuals with the R allele of Sen3 (black), without the R allele (grey) and the proportion of recombinants (black stripes) is given. In (D) are given the Pearson correlations between the resistance mean scores of P2, P6 and P18. Chi square tests were conducted to validate if the resistance to the three pathotypes segregated in a 1:1 ratio in the population (E). [file 13007_2019_445_MOESM5_ESM.docx]

**Additional file 5**

Resistance distribution in the full-sib population (n = 328) for pathotypes 2 (A), 6 (B) and 18 (C). The phenotyping was performed with the Glynne-Lemmerzahl method in 2016. For each resistance category, the proportion of individuals with the R allele of *Sen3* (black), without the R allele (grey) and the proportion of recombinants (black stripes) is given. In (D) are given the Pearson correlations between the resistance mean scores of P2, P6 and P18. Chi-square tests were conducted to validate if the resistance to the three pathotypes segregated in a 1:1 ratio in the population (E).

Kuba

Kuba

(C)

(B)

(A)

| (D) | Pathotype 2 (G1) | Pathotype 6 (O1) | Pathotype 18 (T1) |
| --- | --- | --- | --- |
| Pathotype 2 (G1) | 1 | 0.93 | 0.89 |
| Pathotype 6 (O1) | 0.93 | 1 | 0.91 |
| Pathotype 18 (T1) | 0.89 | 0.91 | 1 |

| (E) | Segregating ratio | χ2 | χ2 test significance |
| --- | --- | --- | --- |
| Pathotype 2 (G1) | 1:1 | 0.30 | ns |
| Pathotype 6 (O1) | 1:1 | 1.48 | ns |
| Pathotype 18 (T1) | 1:1 | 30.49 | *** |

ns = not significant

* : p-value < 0.05

** : p-value < 0.01

*** : p-value < 0.001
