# Supplementary material for: Comparative Subsequence Sets Analysis (CoSSA) is a robust approach to identify haplotype specific SNPs; mapping and pedigree analysis of a potato wart disease resistance gene Sen3
Source: Plant Methods. 2019 May 29;15:60. doi: 10.1186/s13007-019-0445-5 (PMC6540404; doi:10.1186/s13007-019-0445-5)
Supplement: Supplementary file 7 — Additional file 7. Distribution of the number of total k-mers in function of the k-mer depth. The total number of k-mers (unique k-mers x depth) for each sequencing depth from 2 to 100 are represented in this graph for the 4 different samples (red: Kuba, blue: Ludmilla, green: R-bulk, yellow: S-bulk). For the two varieties samples (Kuba and Ludmilla), the peaks of k-mers from simplex, duplex, triplex and quadruplex regions are visible. There is a shift in the simplex peak of the bulks as there are 8 possible haplotypes instead of 4 as in the parental genotypes. [file 13007_2019_445_MOESM7_ESM.docx]

**Additional file 7** The total number of *k*-mers (unique *k*-mers x depth) for each sequencing depth from 2 to 100 are represented in this graph for the 4 different samples (red: Kuba, blue: Ludmilla, green: R-bulk, yellow: S-bulk). For the two varieties samples (Kuba and Ludmilla), the peaks of *k*-mers from simplex, duplex, triplex and quadruplex regions are visible. There is a shift in the simplex peak of the bulks as there are 8 possible haplotypes instead of 4 as in the parental genotypes.

**
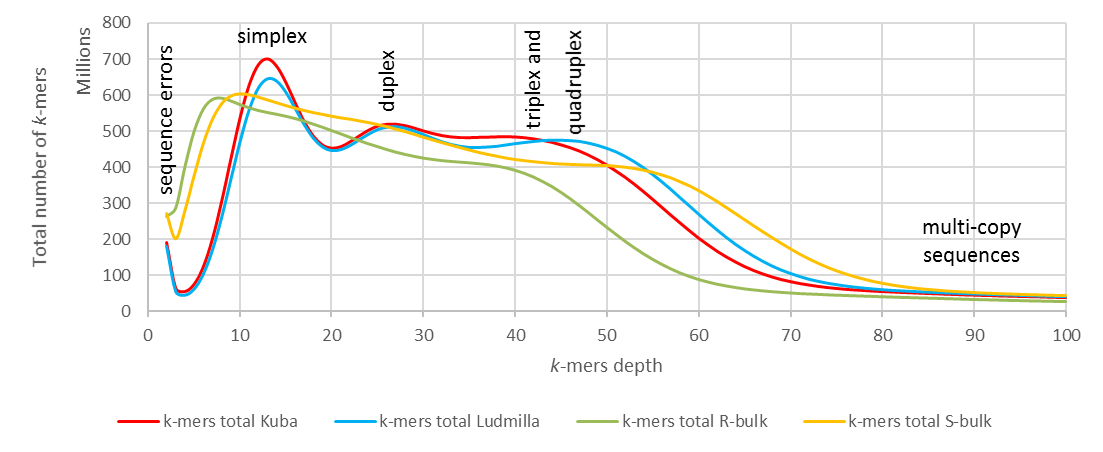
**
