# Supplementary material for: Comparative Subsequence Sets Analysis (CoSSA) is a robust approach to identify haplotype specific SNPs; mapping and pedigree analysis of a potato wart disease resistance gene Sen3
Source: Plant Methods. 2019 May 29;15:60. doi: 10.1186/s13007-019-0445-5 (PMC6540404; doi:10.1186/s13007-019-0445-5)
Supplement: Supplementary file 8 — Additional file 8. CoSSA results with the reference genome. Number of R-bulk specific k-mers (depth 10 to 22) mapping to each 1 Mb bin of (A) chromosome 0, (B) chromosome 3, (C) chromosome 4, (D) chromosome 5, (E) chromosome 9 and (F) chromosome 11 of the potato reference genome DM. Red: k-mers inherited from Kuba (resistance specific k-mers), yellow: k-mers inherited from Kuba minus the k-mers present in the susceptible varieties, blue: k-mers inherited from Ludmilla, green: k-mers inherited from both parents. [file 13007_2019_445_MOESM8_ESM.docx]

**Additional file 8 Number of R-bulk specific *k*-mers (depth 10 to 22) mapping to each 1Mb bin of (A) chromosome 0, (B) chromosome 3, (C) chromosome 4, (D) chromosome 5, (E) chromosome 9 and (F) chromosome 11 of the potato reference genome DM. Red: *k*-mers inherited from Kuba (resistance specific *k*-mers), yellow: *k*-mers inherited from Kuba minus the *k*-mers present in the susceptible varieties, blue: *k*-mers inherited from Ludmilla, green: *k*-mers inherited from both parents.**


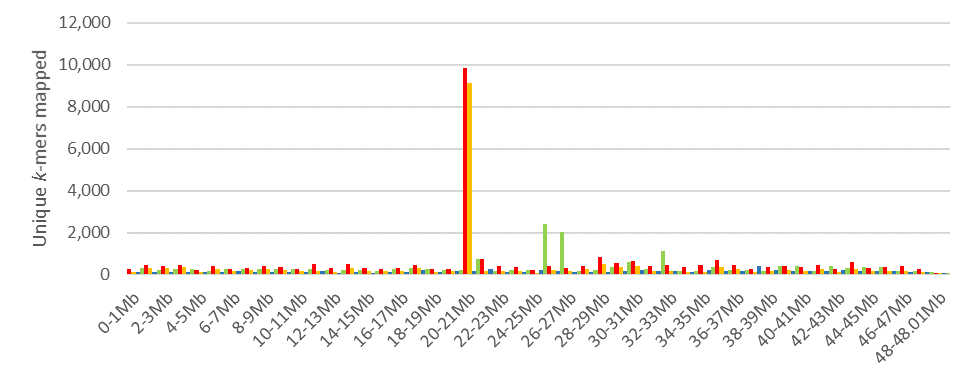


A


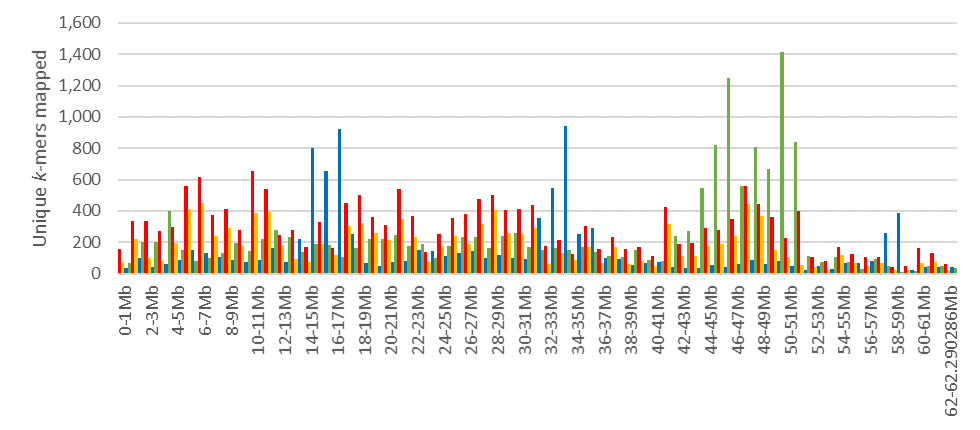


B


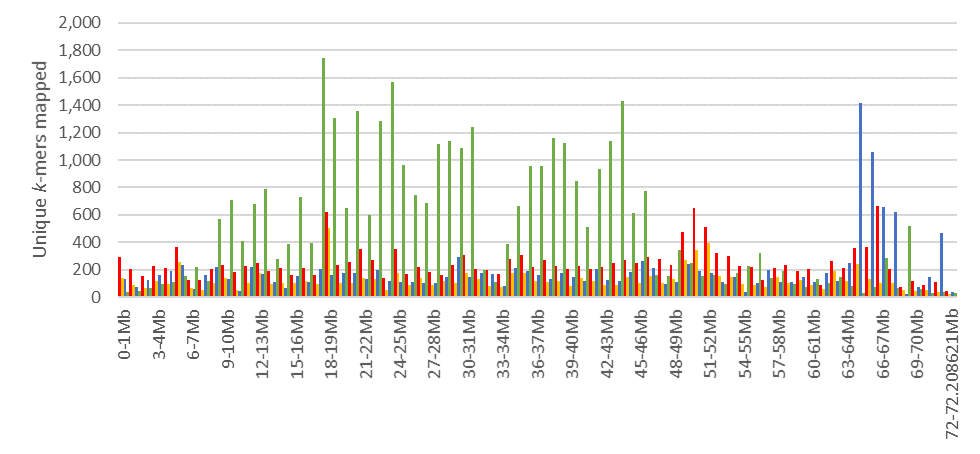


C


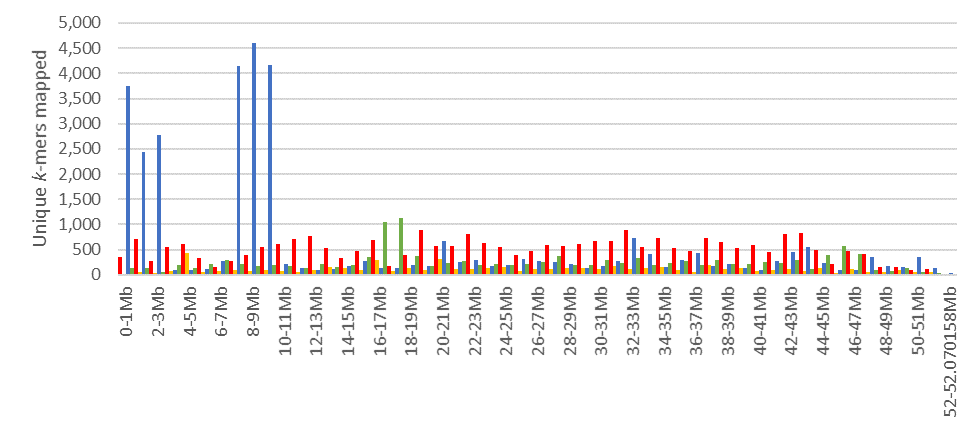


D


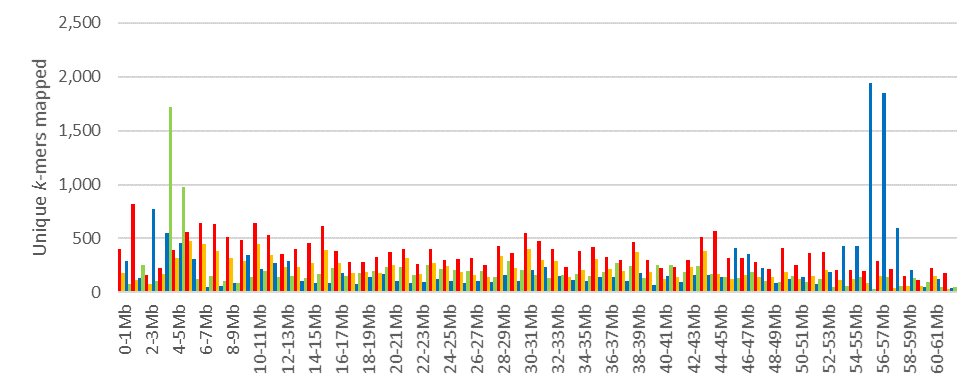


E


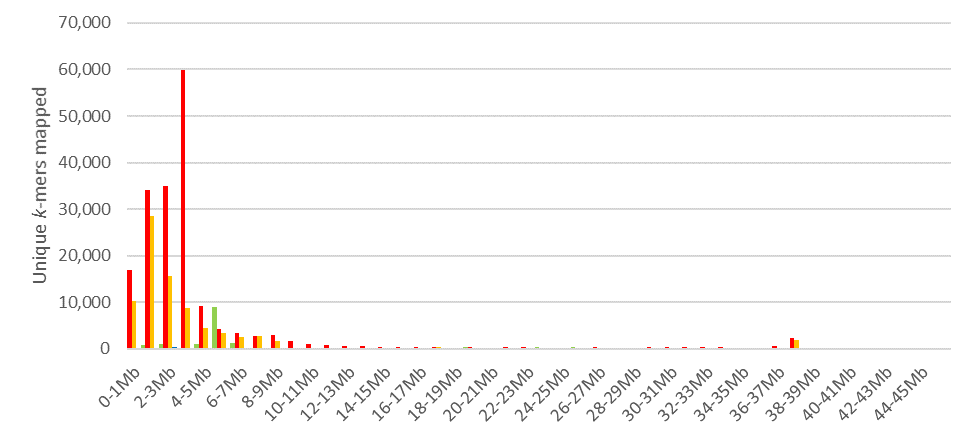


F
