# Supplementary material for: Comparative Subsequence Sets Analysis (CoSSA) is a robust approach to identify haplotype specific SNPs; mapping and pedigree analysis of a potato wart disease resistance gene Sen3
Source: Plant Methods. 2019 May 29;15:60. doi: 10.1186/s13007-019-0445-5 (PMC6540404; doi:10.1186/s13007-019-0445-5)
Supplement: Supplementary file 9 — Additional file 9. Markers effects on pathotypes 2, 6 and 18 resistance. Boxplots of the resistance scores for P2, P6 and P18 in function of the allele (0: S allele, 1: R allele) of the KASP markers chr11_1519485 (A) and chr09_55113777 (B) in the Kuba x Ludmilla population subset (n = 83). [file 13007_2019_445_MOESM9_ESM.docx]

**Additional file 9**

Boxplots of the resistance scores for P2, P6 and P18 in function of the allele (0: S allele, 1: R allele) of the KASP markers chr11_1519485 (A) and chr09_55113777 (B) in the Kuba x Ludmilla population subset (n = 83).


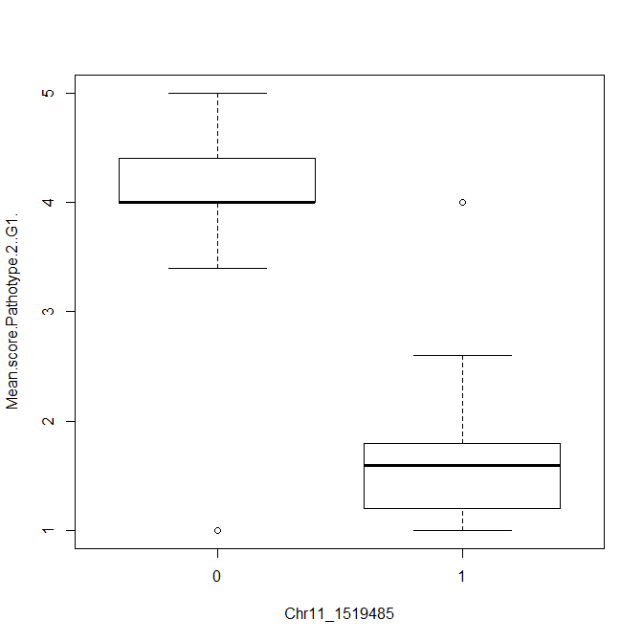

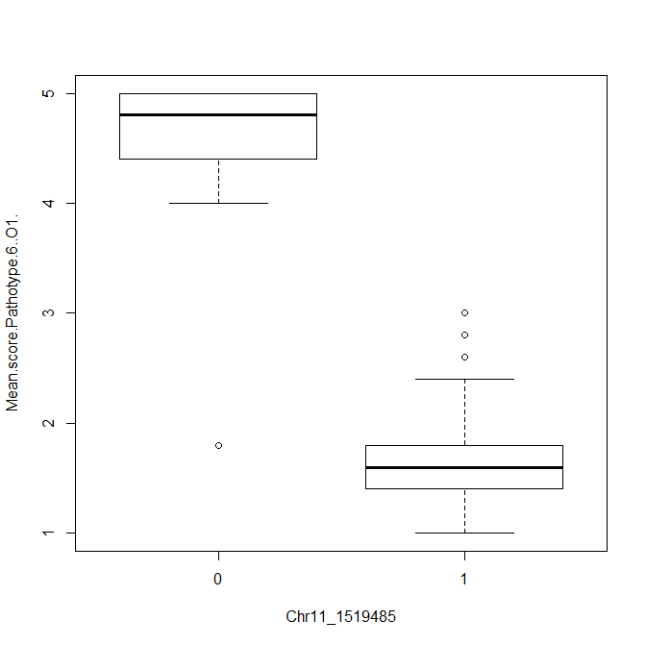


P6

P2


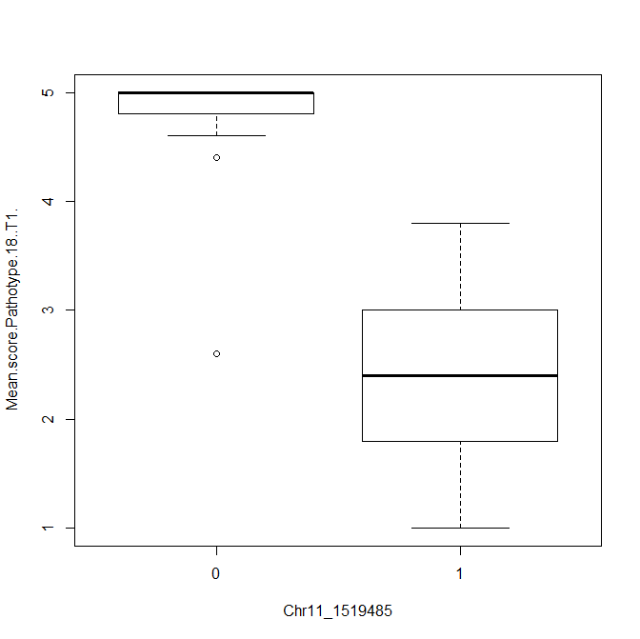


A

P18


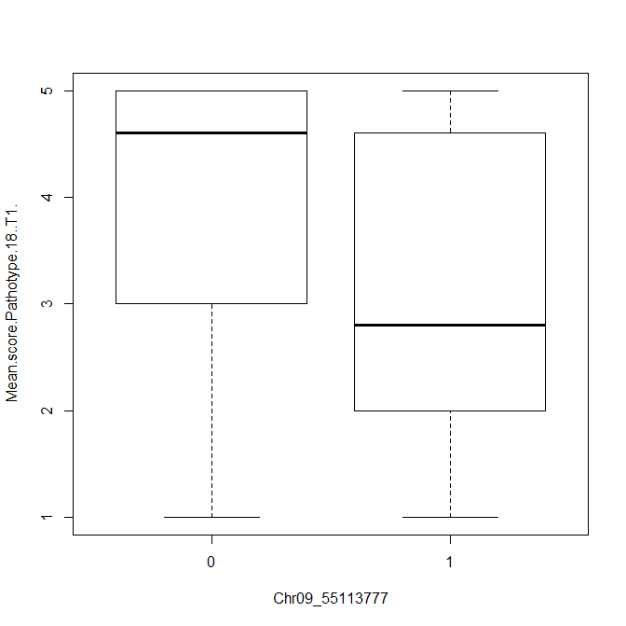

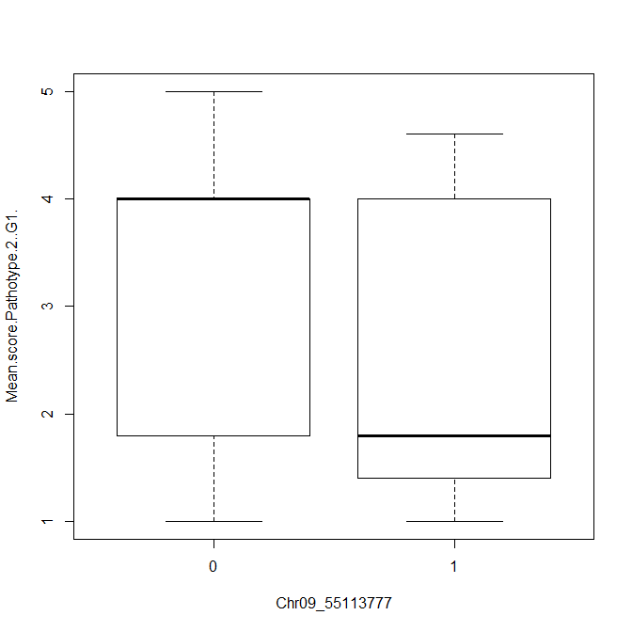

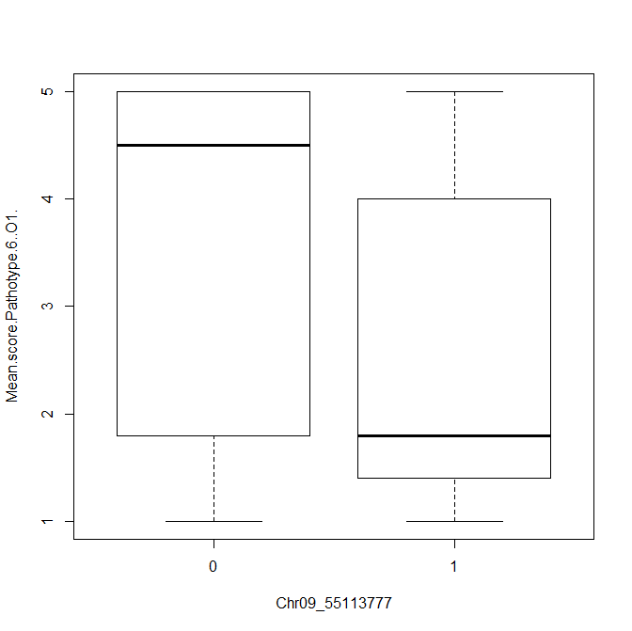


P6

P2

B

P18
