# Supplementary material for: Comparative Subsequence Sets Analysis (CoSSA) is a robust approach to identify haplotype specific SNPs; mapping and pedigree analysis of a potato wart disease resistance gene Sen3
Source: Plant Methods. 2019 May 29;15:60. doi: 10.1186/s13007-019-0445-5 (PMC6540404; doi:10.1186/s13007-019-0445-5)
Supplement: Supplementary file 10 — Additional file 10. De novo assembly statistics. Statistics of the de novo assembly of the resistant haplotype performed. [file 13007_2019_445_MOESM10_ESM.docx]

**Additional file 10**

Statistics of the *de novo* assembly of the resistant haplotype performed.

| Sequencing depth per haploid genome | Number of scaffolds | Longest scaffold (bp) | Mean scaffold size (bp) | N50 |
| --- | --- | --- | --- | --- |
| ~16x | 21,306 | 46,690 | 1,037 | 1,115 |
