# Supplementary material for: Comparative Subsequence Sets Analysis (CoSSA) is a robust approach to identify haplotype specific SNPs; mapping and pedigree analysis of a potato wart disease resistance gene Sen3
Source: Plant Methods. 2019 May 29;15:60. doi: 10.1186/s13007-019-0445-5 (PMC6540404; doi:10.1186/s13007-019-0445-5)
Supplement: Supplementary file 11 — Additional file 11. Comparison of the CoSSA workflows with/without reference. Number and percentage of the k-mers mapping to the three biggest scaffolds of the de novo assembly with 0 mismatches that map to the reference genome (DM) as well. During the mapping process to the reference, 0, 1 and 2 mismatches were allowed. [file 13007_2019_445_MOESM11_ESM.docx]

**Additional file 11**

Number and percentage of the *k*-mers mapping to the three biggest scaffolds of the *de novo* assembly with 0 mismatches that map to the reference genome (DM) as well. During the mapping process to the reference, 0, 1 and 2 mismatches were allowed.

| Scaffold | Length | # of mapped *k*-mers | # of *k*-mers mapped to DM 0 mismatches | # of *k*-mers mapped to DM 1 mismatch | # of *k*-mers mapped to DM 2 mismatches |
| --- | --- | --- | --- | --- | --- |
| NODE_1 | 46,690 | 11,511 | 340 (2,95%) | 2,947 (25.60%) | 5,199 (45,17%) |
| NODE_2 | 44,756 | 8,420 | 49 (0.58%) | 2,788 (33.11%) | 5,118 (60.78%) |
| NODE_3 | 38,969 | 6,629 | 2,774 (41.85%) | 4,417 (66.63%) | 5,426 (81,85%) |
