# Supplementary material for: Comparative Subsequence Sets Analysis (CoSSA) is a robust approach to identify haplotype specific SNPs; mapping and pedigree analysis of a potato wart disease resistance gene Sen3
Source: Plant Methods. 2019 May 29;15:60. doi: 10.1186/s13007-019-0445-5 (PMC6540404; doi:10.1186/s13007-019-0445-5)

**Additional file 12**

The pedigree tree of Kuba (according to (49)) and the known resistance pattern of its ancestors. BRA9089 is present in the pedigree of several varieties resistant to pathotypes 2, 6 and 18 and is thought to be the ancestral donor of their resistance. BRA9089 is present in the resistant grand-parent pedigree (Bzura) and in the susceptible grand-parent (Karlena) pedigree as well.

 
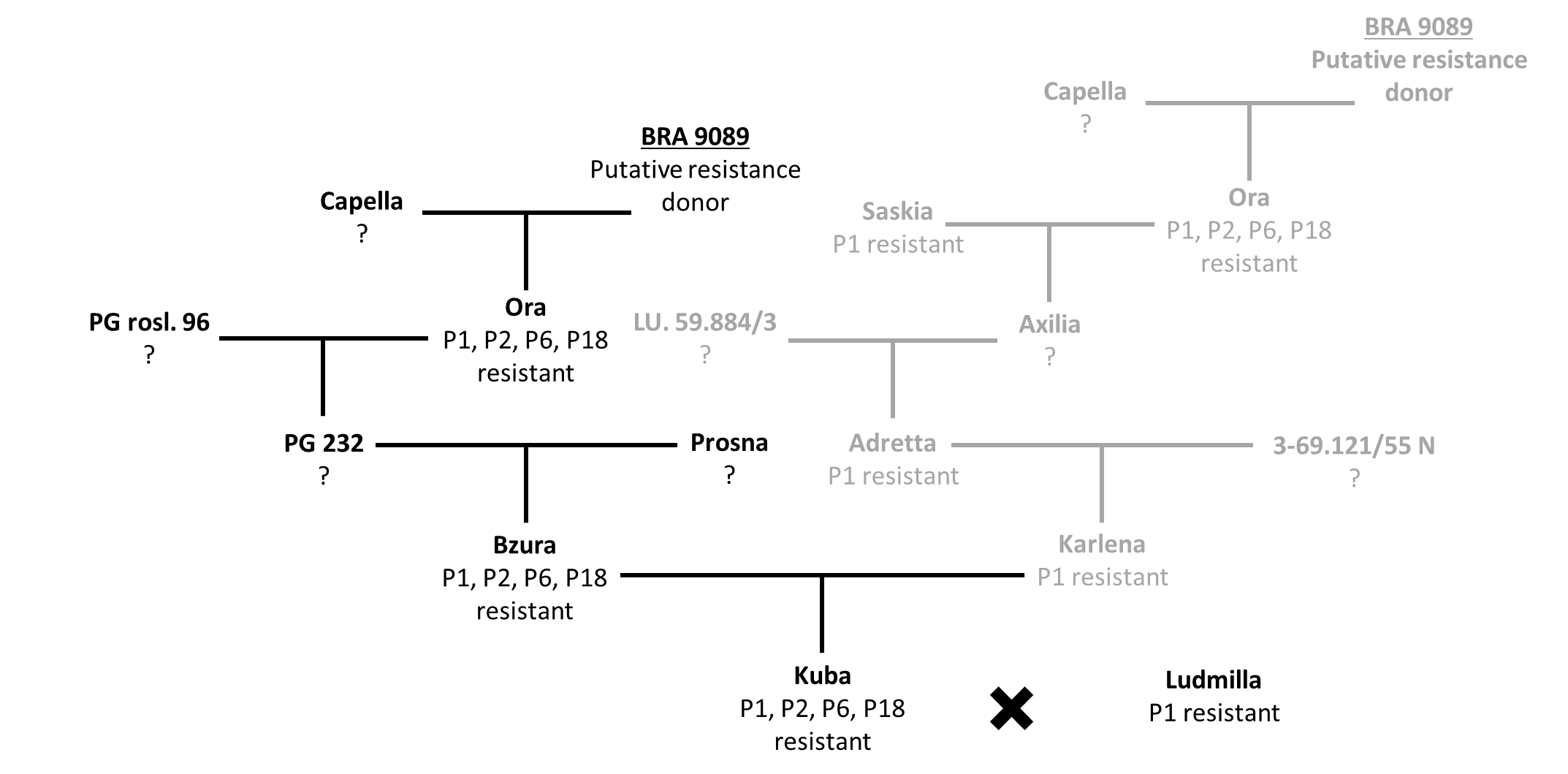

Supplement: Supplementary file 12 — Additional file 12. Pedigree of Kuba. The pedigree tree of Kuba (according to [49]) and the known resistance pattern of its ancestors. BRA9089 is present in the pedigree of several varieties resistant to pathotypes 2, 6 and 18 and is thought to be the ancestral donor of their resistance. BRA9089 is present in the resistant grand-parent pedigree (Bzura) and in the susceptible grand-parent (Karlena) pedigree as well. [file 13007_2019_445_MOESM12_ESM.docx]
