# Supplementary material for: Comparative Subsequence Sets Analysis (CoSSA) is a robust approach to identify haplotype specific SNPs; mapping and pedigree analysis of a potato wart disease resistance gene Sen3
Source: Plant Methods. 2019 May 29;15:60. doi: 10.1186/s13007-019-0445-5 (PMC6540404; doi:10.1186/s13007-019-0445-5)

**Additional file 13**

Number of unique *k*-mers in common between the resistance specific *k*-mers without the S varieties *k*-mers and Bzura (the resistant parent of Kuba), BRA9089 (putative resistant donor) and Kuba, (A) mapped to the reference genome (chromosome 11, 0 - 7Mb, DMB734 *k*-mers were added to the 1-2Mb bin) (B) mapped to the 10 longest scaffolds of the *de novo* assembly. Orange: Kuba, blue: Bzura, black: BRA9089. The bars numbers represent the percentages of the intersection *k*-mers.


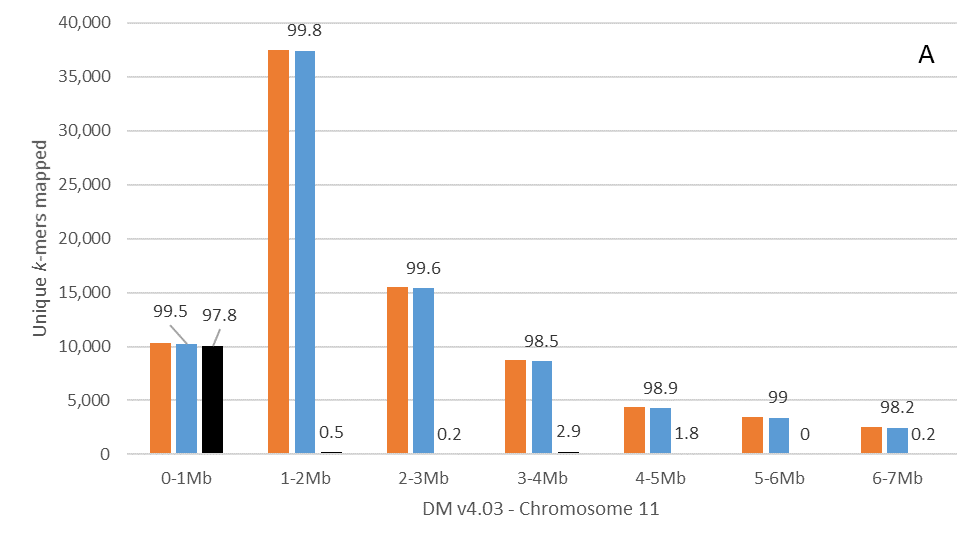

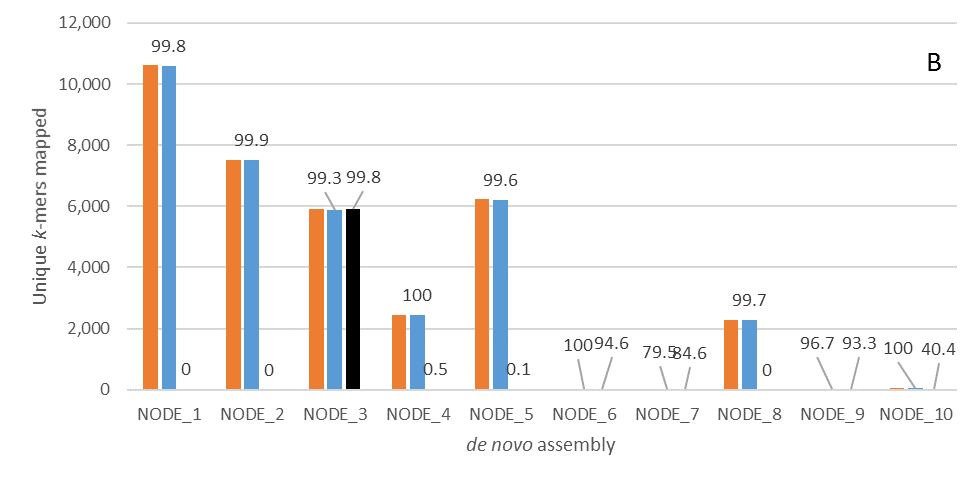

Supplement: Supplementary file 13 — Additional file 13. Pedigree CoSSA analysis. Number of unique k-mers in common between the resistance specific k-mers without the S varieties k-mers and Bzura (the resistant parent of Kuba), BRA9089 (putative resistant donor) and Kuba, (A) mapped to the reference genome (chromosome 11, 0–7 Mb, DMB734 k-mers were added to the 1–2 Mb bin) (B) mapped to the 10 longest scaffolds of the de novo assembly. Orange: Kuba, blue: Bzura, black: BRA9089. The bars numbers represent the percentages of the intersection k-mers. [file 13007_2019_445_MOESM13_ESM.docx]
