# Supplementary material for: Comparative Subsequence Sets Analysis (CoSSA) is a robust approach to identify haplotype specific SNPs; mapping and pedigree analysis of a potato wart disease resistance gene Sen3
Source: Plant Methods. 2019 May 29;15:60. doi: 10.1186/s13007-019-0445-5 (PMC6540404; doi:10.1186/s13007-019-0445-5)
Supplement: Supplementary file 14 — Additional file 14. Assessment of the depth cut-offs on the CoSSA results. Assessment of the effect of the lower and upper depth cut-off on the CoSSA output. (A) CoSSA output without the lower depth cut-off (R-bulk specific k-mers with a depth from 2 to 22 ×), (B) CoSSA output with the lower and upper cut-offs (R-bulk specific k-mers with a depth from 10 to 22 ×), (C) CoSSA output without the upper depth cut-off (R-bulk specific k-mers with a depth from 10 to ∞). Red: k-mers inherited from Kuba (resistance specific k-mers), blue: k-mers inherited from Ludmilla, green: k-mers inherited from both parents, grey: k-mers inherited from none of the parents. (D) Signal to noise ratio (SNR) for Sen3 and Ludmilla_chr9. Black: SNR for CoSSA results when no lower cut-off is applied (2 to 22 ×), stripped: SNR for CoSSA results when an upper and a lower cut-offs are applied (10 to 22 ×), grey: SNR for CoSSA results when no upper cut-off is applied (22 to ∞). [file 13007_2019_445_MOESM14_ESM.docx]

**Additional file 14**

Assessment of the effect of the lower and upper depth cut-off on the CoSSA output. (A) CoSSA output without the lower depth cut-off (R-bulk specific *k*-mers with a depth from 2 to 22x), (B) CoSSA output with the lower and upper cut-offs (R-bulk specific *k*-mers with a depth from 10 to 22x), (C) CoSSA output without the upper depth cut-off (R-bulk specific *k*-mers with a depth from 10 to ∞). **Red: *k*-mers inherited from Kuba (resistance specific *k*-mers), blue: *k*-mers inherited from Ludmilla, green: *k*-mers inherited from both parents, grey: *k*-mers inherited from none of the parents. (D) Signal to noise ratio (SNR) for *Sen3* and *Ludmilla_chr9*. Black: SNR for CoSSA results when no lower cut-off is applied (2 to 22x), stripped: SNR for CoSSA results when an upper and a lower cut-offs are applied (10 to 22x), grey: SNR for CoSSA results when no upper cut-off is applied (22 to ∞).**


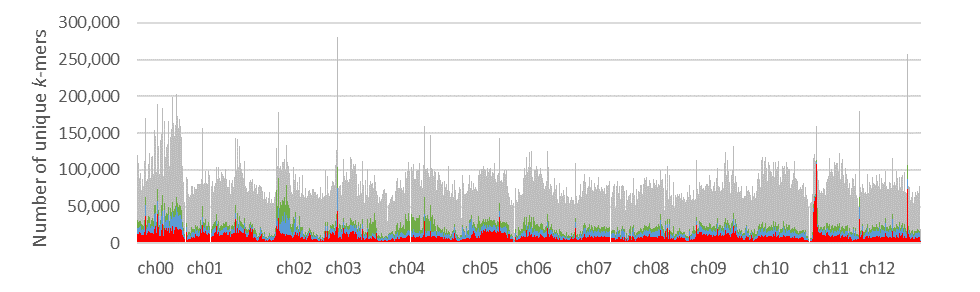


A [2;22x]


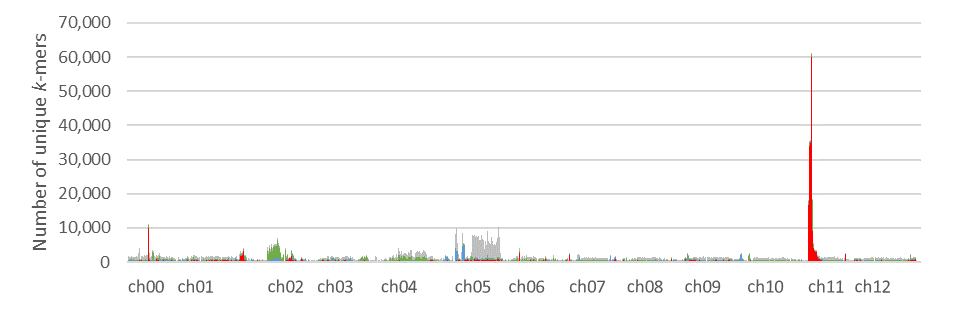


B [10;22x]


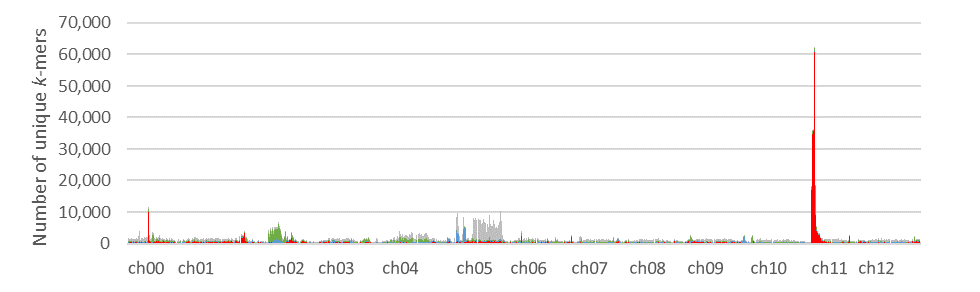


C [10;∞[


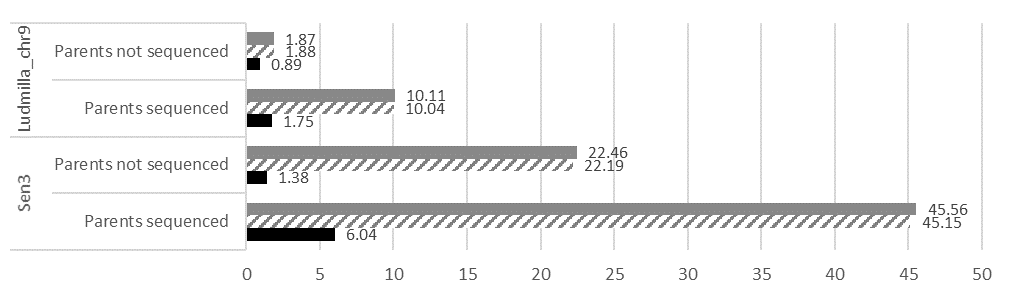


D
