# Supplementary material for: Comparative Subsequence Sets Analysis (CoSSA) is a robust approach to identify haplotype specific SNPs; mapping and pedigree analysis of a potato wart disease resistance gene Sen3
Source: Plant Methods. 2019 May 29;15:60. doi: 10.1186/s13007-019-0445-5 (PMC6540404; doi:10.1186/s13007-019-0445-5)
Supplement: Supplementary file 15 — Additional file 15. Description of the datasets used in the CoSSA simulations with lower sequencing depths. Sequencing depth of the full sequencing data (16 ×/haploid genome) and the two randomly sampled subsets to simulate depths of 10 × and 5 × per haploid genome. For each subset, the k-mers depth cut-off used in the CoSSA is given. [file 13007_2019_445_MOESM15_ESM.docx]

**Additional file 15**

Sequencing depth of the full sequencing data (16x / haploid genome) and the two randomly sampled subsets to simulate depths of 10x and 5x per haploid genome. For each subset, the *k*-mers depth cut-off used in the CoSSA is given.

|  | Sample name | # read pairs | Produced total depth | Depth / haploid genome | *k*-mers cut-off depth |
| --- | --- | --- | --- | --- | --- |
| 16x | R-bulk | 172,190,144 | 61.9 | 15.5 | [10; 22] |
|  | S-bulk | 228,585,110 | 82.2 | 20.5 |  |
|  | Kuba | 200,579,289 | 72.1 | 18.0 |  |
|  | Ludmilla | 195,099,847 | 70.1 | 17.5 |  |
| 10x | R-bulk | 111,258,278 | 40 | 10 | [6; 14] |
|  | S-bulk |  |  |  |  |
|  | Kuba |  |  |  |  |
|  | Ludmilla |  |  |  |  |
| 5x | R-bulk | 55,629,139 | 20 | 5 | [3; 7] |
|  | S-bulk |  |  |  |  |
|  | Kuba |  |  |  |  |
|  | Ludmilla |  |  |  |  |
