# Supplementary material for: Comparative Subsequence Sets Analysis (CoSSA) is a robust approach to identify haplotype specific SNPs; mapping and pedigree analysis of a potato wart disease resistance gene Sen3
Source: Plant Methods. 2019 May 29;15:60. doi: 10.1186/s13007-019-0445-5 (PMC6540404; doi:10.1186/s13007-019-0445-5)
Supplement: Supplementary file 16 — Additional file 16. CoSSA applied with Kuba and the S-bulk only. Kuba specific k-mers (depth 8 × to 18 ×) obtained by the difference between Kuba and the S-bulk mapped to the reference genome. [file 13007_2019_445_MOESM16_ESM.docx]

**Additional file 16**

Kuba specific *k*-mers (depth 8x to 18x) obtained by the difference between Kuba and the S-bulk mapped to the reference genome.
